# Transcriptional Profiling Reveals the Wheat Defences against Fusarium Head Blight Disease Regulated by a NAC Transcription Factor

**DOI:** 10.3390/plants12142708

**Published:** 2023-07-20

**Authors:** Monika Vranić, Alexandre Perochon, Fiona M. Doohan

**Affiliations:** UCD School of Biology and Environmental Science and Earth Institute, College of Science, University College Dublin, D04 V1W8 Dublin, Ireland

**Keywords:** cereal, defence, deoxynivalenol, disease resistance, Fusarium head blight, NAC transcription factor, wheat

## Abstract

The wheat NAC transcription factor TaNACL-D1 enhances resistance to the economically devastating Fusarium head blight (FHB) disease. The objective of this study was to decipher the alterations in gene expression, pathways and biological processes that led to enhanced resistance as a result of the constitutive expression of TaNACL-D1 in wheat. Transcriptomic analysis was used to determine the genes and processes enhanced in wheat due to TaNACL-D1 overexpression, both in the presence and absence of the causal agent of FHB, *Fusarium graminearum* (0- and 1-day post-treatment). The overexpression of TaNACL-D1 resulted in more pronounced transcriptional reprogramming as a response to fungal infection, leading to the enhanced expression of genes involved in detoxification, immune responses, secondary metabolism, hormone biosynthesis, and signalling. The regulation and response to JA and ABA were differentially regulated between the OE and the WT. Furthermore, the results suggest that the OE may more efficiently: (i) regulate the oxidative burst; (ii) modulate cell death; and (iii) induce both the phenylpropanoid pathway and lignin synthesis. Thus, this study provides insights into the mode of action and downstream target pathways for this novel NAC transcription factor, further validating its potential as a gene to enhance FHB resistance in wheat.

## 1. Introduction

NAC (No apical meristem (NAM), *Arabidopsis thaliana* transcription activation factor (ATAF1/2) and cup-shaped cotyledon (CUC2)) transcription factors (TFs) represent one of the largest plant families of transcriptional regulators. They are delineated by their conserved NAC domains containing five subdomains, A-E, of which A-D form the NAM domain [1]. The NAC domains are usually situated in the N-terminal part of the protein and are associated with DNA-binding, transcriptional control, and homo- and heterodimerization [2]. NAC proteins also encode a highly divergent transcriptional activation region (TAR) in the C-terminal part of the protein associated with transcriptional activation and protein–protein interactions [2]. Many studies have elucidated the importance of NAC TFs in plant defence against pathogens. Members of this TF family regulate host basal defences via the direct or indirect induction of defence-related genes such as salicylic acid (SA)-related pathogenesis-related (PR) genes, jasmonic acid (JA)-related plant defensins (PDF); defence-associated TFs such as WRKY; or by modulating the hypersensitive response (HR)-induced cell death [3,4,5,6]. NACs have been shown to mediate crosstalk between different hormonal pathways. For example, the well-studied tomato SlNAP1 mediated a crosstalk between SA, gibberellic acid (GA) and abscisic acid (ABA) signalling pathways to regulate growth and abiotic/biotic stress responses [5]. NACs can enhance plant resistance to pathogens by modulating the biosynthesis of antimicrobial phytoalexins [7]. A microarray study of rice plants overexpressing defence-associated OsNAC6 elucidated that NAC-regulated genes were involved in detoxification, redox homeostasis, proteolysis, and defence-associated proteins such as β-1,3-glucanase-like and chitinases [8]. Also, NACs can regulate the phenylpropanoid pathway and lignin-biosynthetic genes. For example, wheat TaNAC032 and lily LrNAC35 positively regulated the lignification of cell walls in response to *Fusarium graminearum* infection and viral attack, respectively [9,10].

*Fusarium graminearum* is the causal agent of the economically devastating Fusarium head blight (FHB) disease of wheat and other small grain cereals. FHB results in yield loss and the mycotoxin contamination of grains [11]. It infects wheat heads during flowering, with a short symptomless biotrophic phase of infection preceding a necrotrophic phase of disease [12,13,14]. The mycotoxin deoxynivalenol (DON) is produced by the fungus to facilitate the spread of the fungus through the rachis and to adjacent spikelets and grains. Many wheat NAC genes (TaNACs) are responsive to diseases, as recently described [15], and several have been associated with either susceptibility or resistance to FHB. TaNACs were associated with enhanced FHB susceptibility based on transcriptional profiling of resistant and susceptible wheat cultivars [16]. Wheat NAC secondary wall thickening-promoting factor 1 like (NST1-like) was determined to be a potential candidate for the wheat genomic locus (Qfhs.ifa-5Ac) associated with FHB resistance [17]. Recently, TaNACL-D1 and TaNAC032 were characterised as positive regulators of FHB resistance [9,18]. TaNAC032 is a member of the NAC subfamily ‘a’, which is enriched with pathogen-responsive TaNACs, while TaNACL-D1 was placed in the evolutionary distant subfamily ‘h’ of NACs which was not previously associated with defence [15].

Very few studies have investigated the transcriptional/metabolomic reprogramming associated with overexpression/silencing of defence-associated NAC TFs. This leaves a large gap in our understanding of the exact molecular mechanisms behind NAC-mediated pathogen defence. In this study, RNA sequencing was employed to investigate the impact of *TaNACL-D1* overexpression on the response of the wheat transcriptome to *F. graminearum*, and to unravel the defence-associated pathways and key genes that potentially led to the enhanced FHB resistance associated with overexpression of this gene [18]. The study compared the early transcriptomic response to *F. graminearum* of spikelets of a *TaNACL-D1*- overexpressing line with that of the wild type wheat cultivar (cv.) Fielder. The study focused on the early stage of infection (one day post-inoculation), corresponding to the late biotrophic phase or switch to the necrotrophic phase. Gene ontology and KEGG (Kyoto Encyclopaedia of Genes and Genomes) analysis of genes differentially expressed between wild type and the overexpressing genotype identified up/downregulated biological processes and pathways that are likely involved in NAC-mediated FHB resistance.

## 2. Results

### 2.1. Validation of Enhanced FHB Resistance in the TaNACL-D1 Overexpressing Line (OE-2)

**Figure 1:**
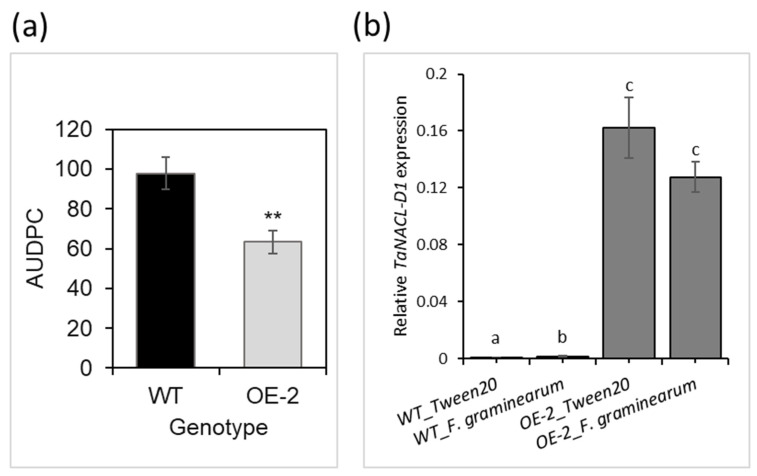
(**a**) Effect of TaNACL-D1 overexpression in the TaNACL-D1-overexpressing line (OE-2, grey column) derived from the wheat cv. Fielder on Fusarium head blight resistance, as compared to the wild type (WT, black column). At mid-anthesis, central flowering spikelets from the WT and OE-2 were point-inoculated with *Fusarium graminearum* strain GZ3639. Disease was assessed at different days post-inoculation (dpi) and data presented correspond to the area under the disease progress curve (AUDPC). Results represent the mean of the three trials and error bars indicate ± SEM (*n* = 30–31). The asterisks indicate a significant difference, as compared to the WT (Mann-Whitney *U* test; ** *p* < 0.01); and (**b**) TaNACL-D1 transcript levels in wheat heads after treatment with *F. graminearum* assessed via qRT-PCR at one day post-inoculation. TaPP2AA3 and TaYLS8 housekeeping genes were used as internal reference to calculate the relative expression of TaNACL-D1 using the formula 2−(Ct target gene−Ct average housekeeping genes). Wheat cv. Fielder spikelets were treated with either wild type *F. graminearum* strain GZ3639 or Tween20 (mock). Results represent the mean of three trials and error bars indicate ± SEM (*n* = 12). Different letters above columns indicate significant differences between conditions (Kruskal–Wallis test; *p* < 0.05).

As part of the RNA-seq study, the enhanced phenotypic FHB resistance of the TaNACL-D1- overexpressing line OE-2, as compared to the wild type (WT) cv. Fielder, was validated. The area under the disease progression curve (AUDPC) was calculated using disease scores from 7, 14 and 21 dpi. The AUDPC was significantly lower (35% lower) for the OE-2 line as compared to the WT plants (Figure 1a). Thus, the experiment validated that TaNACL-D1 overexpression enhanced resistance against the FHB. qRT-PCR analysis confirmed overexpression of TaNACL-D1 in the OE-2 line at the same level upon treatment with the fungus and mock, as compared to very low to no expression of the gene in the WT upon both treatments (Figure 1b).

### 2.2. RNA-Sequencing

A cDNA library was generated and sequenced for each RNA sample (see Table S1 for reads and genome mapping data). Between 48 to 50 million clean reads of 100 bp length were obtained for each cDNA library. Of these, >83% mapped to the *F. graminearum* and *T. aestivum* pangenome (312919 *F. graminearum* and wheat cDNA sequences), with >52% mapping to a unique target sequence. For each library, >96% of the clean reads had Q20 quality scores (a probability of an error in base calling of 1%). For wheat, there were 75,014 transcripts expressed across the samples, corresponding to 64,658 expressed genes (average of 1.16 transcript variants per expressed gene). As illustrated in Figure S1, expressed transcripts were evenly distributed among wheat subgenomes, with 33, 32 and 33% of transcripts mapping to A, B and D, respectively (1.3% assigned to an unknown subgenome/chromosome U). Chromosomes 2A, 2B, 2D, 3A, 3B, 3D, 5A, 5B and 5D had the highest proportion of expressed transcripts (each accounting for 16% of all wheat transcripts). The total number of expressed transcripts was relatively equal across samples, with an average of 64,268 expressed transcripts per sample (treatment x genotype combination). The Pearson correlation of gene expression between the three trials was significant and strong (>0.9, *p* < 0.05). Principal component analysis (PCA) revealed that gene expression in the samples was mainly driven by the *F. graminearum* vs. mock (Tween 20) treatment, accounting for 86% of the variation in the first principal component. The proportion of *F. graminearum*-expressed transcripts was higher in the pangenome of the overexpressing line (0.098%) as compared to the WT (0.088%). As expected, except for one (most likely misaligned) transcript, no fungal transcripts were detected in non-treated/Tween20 treated control samples. Overall, the transcriptome results reported herein indicated quality RNA transcriptome data suitable for differential expression analysis.

### 2.3. The Effect of TaNACL-D1 Overexpression and Fusarium Treatment on the Transcriptome of Wheat

**Figure 2:**
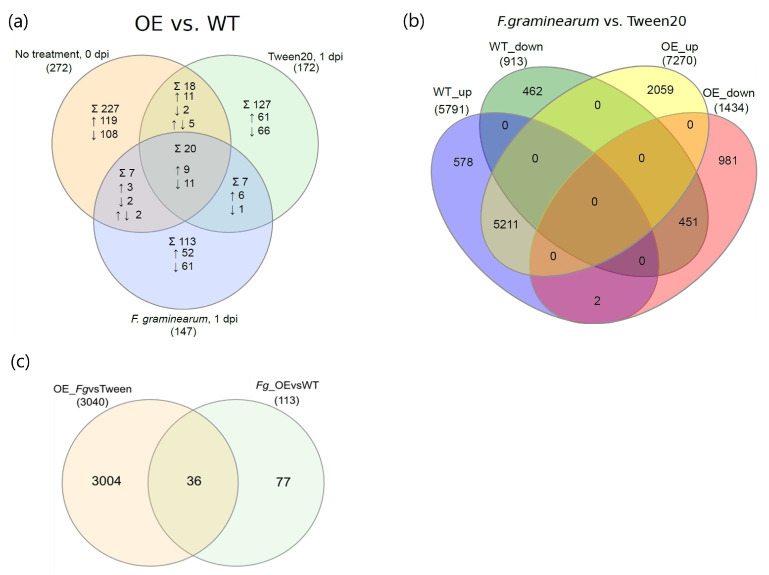
Venn diagrams illustrating the impact of TaNACL-D1 overexpression and *F. graminearum* inoculation on the wheat transcriptome: (**a**) TaNACL-D1-regulated transcript numbers (differentially expressed between the overexpressor (OE) and the wild type (WT) in either non-treated plants (day zero), Tween20-treated plants (mock) at one-day post-inoculation (dpi), or *F. graminearum*-treated plants at 1 dpi; (**b**) *F. graminearum* (Fg)-regulated transcripts (differentially expressed between Fg and mock treatment) in the WT and OE at 1 dpi; and (**c**) Venn diagram illustrating that 36 transcripts were both responsive to *F. graminearum* in the OE (and not in the WT) and significantly more- or less-expressed (higher or lower transcript levels) in the pathogen-treated OE versus WT spikelets (at 1 dpi). In each Venn diagram, the numbers in brackets indicate the total number of transcripts in each comparison group, the numbers in the overlapping areas indicate those being shared between two or more comparison groups, and the numbers in the areas not being shared by any of the comparison groups indicate those transcripts specific to the group. Symbols and abbreviations: ↑ Upregulated (up); ↓ Downregulated (down); ↑↓ Up- and downregulated; ∑ total number of TaNACL-D1-regulated transcripts.

Differential expression analysis compared the effect of TaNACL-D1 gene overexpression on the wheat transcriptome of cv. Fielder, both in the absence and presence of the pathogen (Table S2 and Figure 2a). At 0 dpi, 1-day post-mock and 1-day post-*Fusarium* treatment, there were, respectively, 272, 172 and 147 transcripts differentially regulated by TaNACL-D1 overexpression in the OE as compared to WT (Figure 2a). Differential expression analysis also assessed the effect of *Fusarium* treatment on transcriptomes of the TaNACL-D1 overexpressor as compared to the WT (Table S2 and Figure 2b). A total of 9744 transcripts were *Fusarium-*responsive, of which 58% were common to both the OE and WT, 32% were specific for the OE, and 11% to the WT (Figure 2b). There were 2000 more up/downregulated *Fusarium*-regulated transcripts (representing 1870 *Fusarium-*regulated genes) in the OE compared to the WT (Table S2, Figure 2b), thus indicating more induced transcriptional reprogramming upon infection in the OE as compared to the WT.

### 2.4. An Overview of the Biological Processes Modulated by F. graminearum Infection in TaNACL-D1 Overexpressing and Wild Type cv. Fielder

The 8704 and 6704 *F. graminearum*-responsive transcripts in the OE and WT, respectively, were analysed, both in terms of gene ontology (GO) categorisation and putative function. Based on the GO annotation, in the OE and WT, respectively, 358 and 315 biological processes were associated with pathogen-upregulated transcripts (299 common to both genotypes) and 169 and 36 with pathogen-downregulated transcripts (29 common to both genotypes) (Table S3). Thus, most of the upregulated biological processes impacted by *F. graminearum* were common between genotypes, while most downregulated biological processes were specific to the OE. The number of *F. graminearum*-responsive transcripts in the OE and WT that map to biological processes previously associated with defence against *F. graminearum* (hormone biosynthesis and response, detoxification and phenylpropanoid biosynthesis [19]) are detailed in Table S4. Associated descent molecular functions regulated by the fungus in the OE and WT are illustrated in Figure S4).

### 2.5. Genotype-Specific F. graminearum-Responsive Biological Processes

**Figure 3:**
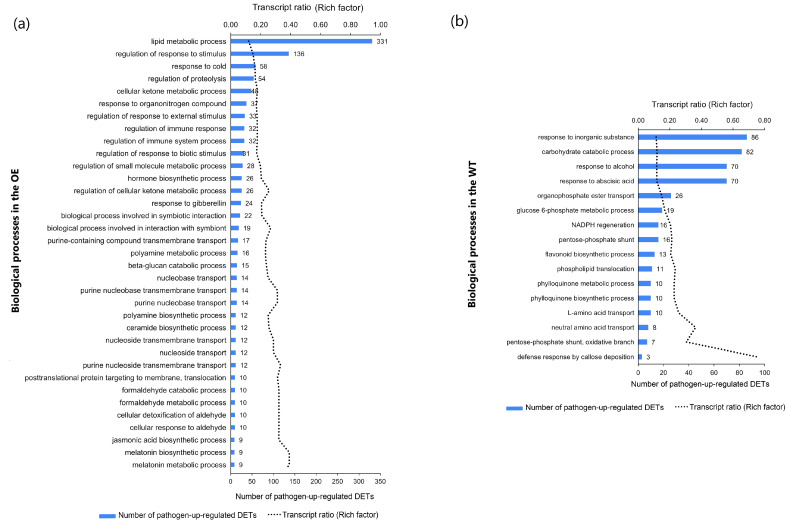
Biological processes enriched in pathogen-upregulated transcripts in: (**a**) the TaNACL-D1-overexpressing (OE) wheat cv. Fielder (but not in the wild type) (*n* = 35); and (**b**) the wild-type cv. Fielder (but not in the TaNAC overexpressor OE) (*n* = 16). The number of pathogen-upregulated transcripts and the transcript ratio associated with each of these biological processes is shown and biological processes are listed based on the number of transcripts (largest to smallest number). Abbreviations: DETs, differentially expressed transcripts.

GO analysis of pathogen-upregulated transcripts highlighted 59 biological processes that were specific to TaNACL-D1-overexpressing cv. Fielder (OE). The top 35 of these, in terms of enrichment in pathogen-responsive transcripts, are illustrated in Figure 3a and were mainly related to ‘metabolism’, ‘biosynthesis’, ‘regulation of response to stimulus’ and ‘transport’. Of all the OE-specific processes, the ‘lipid metabolic process’ was the most enriched in pathogen-upregulated transcripts. The ‘JA biosynthetic process’ was previously associated with defence against FHB [20] and it was enriched in pathogen-upregulated transcripts only in the OE. Molecular functions associated with JA biosynthesis were also explored, revealing that pathogen-upregulated transcripts associated with ‘linoleate 13S lipoxygenase activity’ and ‘12−oxophytodienoate reductase activity’ were enriched only in the OE (Figure S4). Sixteen of the biological processes enriched in pathogen-upregulated transcripts were unique to the WT (Figure 3b), and they were mainly related to ‘metabolism’, ‘transport’, and ‘response to endogenous stimulus’. They included processes previously associated with FHB susceptibility: response to ABA [16,21], and ‘indole-3-acetic acid (IAA) amido synthetase activity’ [20]. Also, the ‘pentose–phosphate shunt’ and the related ‘glucose 6–phosphate metabolic process’ were enriched only in the WT, and these metabolic processes involved in the production of nicotinamide adenine dinucleotide phosphate (NADPH) were previously shown to be upregulated in cultivars susceptible, but not in those resistant. to *F. graminearum* [20].

**Figure 4:**
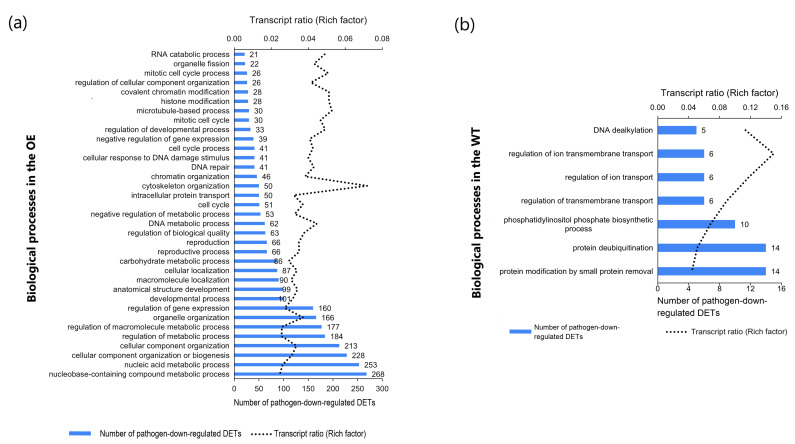
Biological processes enriched in pathogen-downregulated transcripts in: (**a**) the TaNACL-D1-overexpressing wheat cv. Fielder (but not in the wild type) (*n* = 35); and (**b**) the wild-type wheat cv. Fielder (but not in the TaNAC overexpressor OE) (*n* = 7). The number of pathogen-downregulated transcripts and the transcript ratio associated with each of these biological processes is shown and biological processes are listed based on the number of transcripts (smallest to largest number). Abbreviations: DETs, differentially expressed transcripts.

One hundred and forty biological processes were enriched in pathogen-downregulated transcripts in the OE, but not the WT, and the top 35 of these (in terms of the enrichment in pathogen-responsive transcripts) are illustrated in Figure 4a. They are essential primary metabolic and developmental processes. Within the GO term ‘developmental process’, descendent processes unique to the OE were: ‘pollen development’, ‘positive regulation of embryonic development’ and ‘meristem maintenance’ (Figure S4). ‘TORC1 (Target of Rapamycin Complex I) signalling’ is a master regulator of developmental processes [22] and this ‘cellular process’ was enriched in pathogen-downregulated transcripts only in the OE. The cytoskeleton is known to play an important role in pathogen defence [23], but its role in defence against *F. graminearum* has not been investigated. Many of the pathogen-downregulated cytoskeleton-associated processes were unique to the OE, such as ‘microtubule and actin filament binding’, ‘regulation of actin filament polymerization’ and ‘microtubule-based movement’ (Figure S3). Interestingly, ‘positive regulation of ABA biosynthetic process’ was pathogen-downregulated only in the OE (Figure S3). As mentioned earlier, ‘response to ABA’ was pathogen-upregulated only in the WT. Thus, results indicated suppressed and induced ABA-mediated responses to the pathogen in the OE and WT, respectively. Regarding the WT, the seven biological processes enriched in pathogen-downregulated transcripts only in the WT are illustrated in Figure 4b, and they were related to ‘transmembrane transport’, ‘protein modification’ and ‘phosphatidylinositol phosphate biosynthetic process’.

### 2.6. Assessing the Genotype-Associated Transcriptional Differences within Pathogen-Regulated Biological Processes Common to the OE and WT

**Figure 5:**
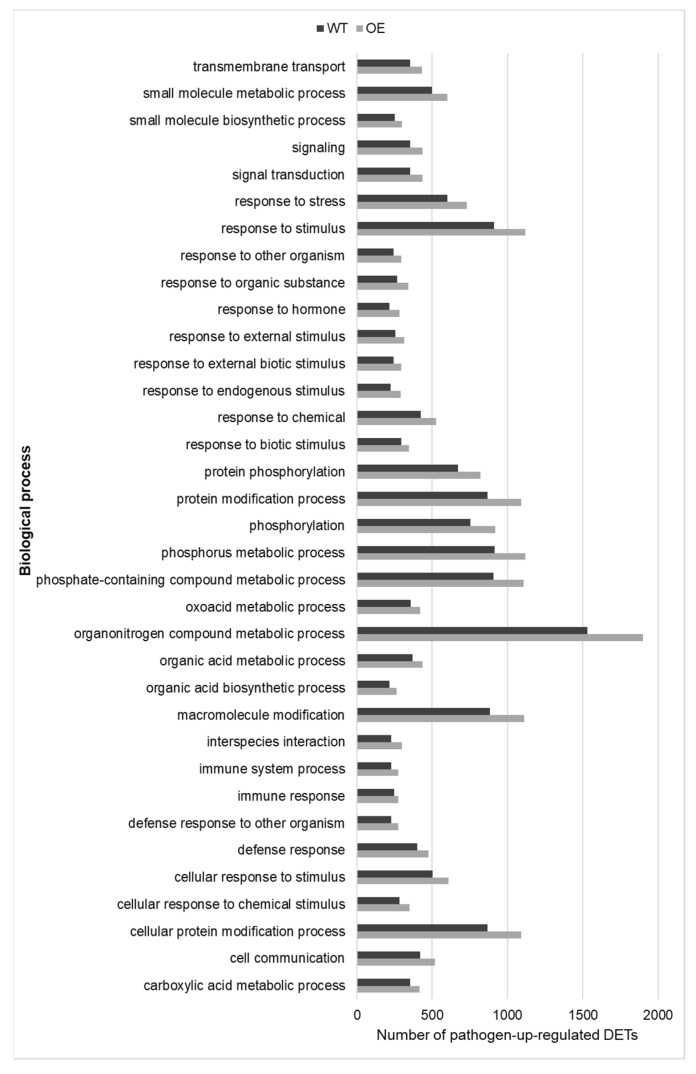
Biological processes (*n* = 35) enriched in *TaNACL-D1*-overexpressing (OE) wheat cv. Fielder and wild type, based on pathogen-upregulated transcripts. The number of pathogen-upregulated transcripts associated with each of these biological processes is shown and biological processes are listed in an alphabetic order.

Three hundred and twenty-eight pathogen-responsive biological processes were common to both genotypes. Analysis was conducted to determine if the magnitude of their response differed between the OE and WT. A genotype was considered more pathogen-induced within a biological process if the number of associated pathogen-regulated transcripts and transcript ratios (Rich factor) were higher than the other genotype. A total of 299 pathogen-upregulated processes were common to both genotypes and analysis focused on the 35 that were most enriched in pathogen-upregulated transcripts, as illustrated in Figure 5. The OE had, on average, 1.2-fold more pathogen-upregulated transcripts and 1.2 -fold higher transcript ratio associated with each of the 35 common biological processes, as compared to the WT (Figure 5, Table S5). These 35 biological processes were related to ‘metabolism’, ‘response to external stimulus’, ‘response to endogenous stimulus’, ‘response to biotic stimulus’, ‘response to stress’, ‘cell communication’, ‘protein metabolism’, ‘biosynthesis’, ‘transport’ and ‘signal transduction’. Processes related to pathogen defence had more pathogen-upregulated transcripts in the OE compared to the WT. For example, the number of pathogen-upregulated transcripts and the transcript ratio was ≥1.2 in the OE versus the WT for ‘biotic stimulus’, ‘defence response’, ‘immune response’ and ‘response to hormones’ (Figure 5, Table S5).

Hormone pathways are known to play a major role in defence against *F. graminearum* (as reviewed by Kazan and Gardiner, [19]). Thus, the differences between genotypes in hormone-related processes responsive to the fungus were explored in more detail (Table S4). Relative to the WT, the OE had between 1.1 and 1.5-fold more pathogen-upregulated transcripts and 1.1–1.4-fold higher transcript ratios for processes associated with oxylipin biosynthesis (which includes JA), salicylic acid (SA), gibberellic acid (GA), response to ethylene (ET) and the ET-activated signalling pathway (Figures S2 and S4, Table S4). Molecular functions associated with ‘methyl jasmonate esterase activity’, ‘methyl salicylate esterase activity’ ‘negative regulation of gibberellic acid-mediated signalling pathway’, and ‘indole-containing compound metabolic and biosynthetic processes’ were more pronounced in the OE as compared to the WT (1.1–1.4-fold more pathogen-upregulated transcripts and 1.1–1.4-fold higher transcript ratio (Figures S2 and S4, Table S4).

**Figure 6:**
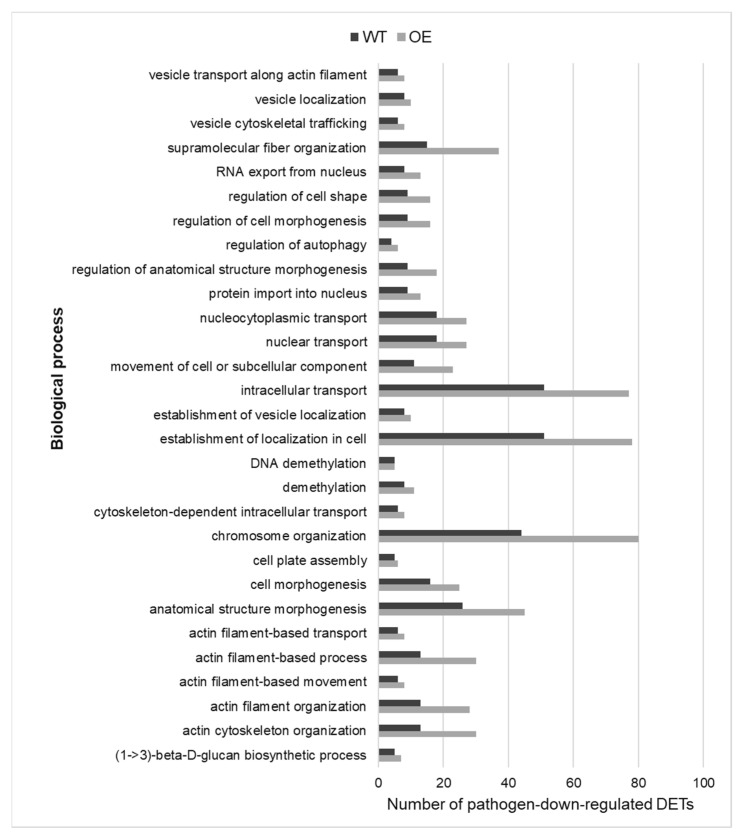
Biological processes (*n* = 29) enriched in TaNACL-D1-overexpressing (OE) wheat cv. Fielder and wild type, based on pathogen-downregulated transcripts. The number of pathogen-downregulated transcripts associated with each of these biological processes is shown and biological processes are listed in an alphabetic order.

A total of 29 pathogen-downregulated biological processes were common to both the OE and WT (Figure 6). These related to ‘transport’, ‘cell organization and biogenesis’, ‘cell morphogenesis’, ‘metabolic process’, ‘organelle/cytoskeleton organization and biogenesis’ and ‘movement of cell or subcellular component’. The OE had on average 1.6-fold more pathogen-downregulated transcripts and 1.6-fold higher transcript ratio associated with each of these common biological processes, as compared to the WT (Figure 6; Table S6). The results indicated that *F. graminearum* affected developmental and primary metabolism-related processes to a higher extent in the OE as compared to the WT, possibly to relocate energy to defence-related processes.

### 2.7. F. graminearum-Responsive Detoxification and Phenylpropanoid Biosynthesis Processes Modulated by TaNACL-D1 Overexpression

Biological processes and molecular functions associated with the phenylpropanoid pathway play an important role in resistance to *F. graminearum* [19,24,25] and thus were explored in more detail. The OE had on average 1.1-fold more pathogen-upregulated transcripts and 1.1-fold higher transcript ratio associated with ‘phenylpropanoid metabolic/biosynthetic process’, as compared to the WT (Table S4). This included transcripts associated with the descendent ‘lignin biosynthetic process’, ‘cinnamic acid biosynthetic process’, ‘phenylalanine ammonia lyase activity’, ‘cinnamyl-alcohol dehydrogenase activity’ and ‘L-phenylalanine catabolic process’ (Figures S2 and S4a). These results indicate a more pronounced pathogen induction of the phenylpropanoid pathway in the OE compared to the WT. Several enzymes involved in a shunt phenylpropanoid pathway were previously determined to be associated with cell-wall reinforcement as a response to *F. graminearum* or its mycotoxin DON [24,26]. Here, transcripts coding for these enzymes, including coumaroyltransferase, amine specific hydroxycinnamoyltransferase, caffeic acid O-methyltransferase and caffeoyl-CoA O-methyltransferase were generally pathogen-upregulated in the OE at a higher level as compared to the WT (Table S7). Also, transcript coding for TaWRKY70 that enhanced resistance against *F. graminearum* via regulation of resistance-related metabolite biosynthetic genes implicated in cell-wall enforcement [27] was pathogen-upregulated at a higher level in the OE as compared to the WT (Table S7). Thus, the OE underwent more pronounced pathogen-upregulation of transcripts associated with cell-wall reinforcement during the response to *F. graminearum,* as compared to the WT.

The alleviation of oxidative stress protects the cell from oxidative damage and the detoxification of mycotoxins produced by the fungus [19,28]. Thus, associated processes/functions were explored in more detail. The OE had on average of 1.1-fold more pathogen-upregulated transcripts and a 1.1-fold higher transcript ratio associated with detoxification-related biological processes (Table S4). Within this process, the OE was more enriched than the WT in associated ‘glutathione metabolic process’, ‘hydrogen peroxide catabolic process’ and ‘response to oxidative stress’ (1.1–1.2-fold more pathogen-upregulated transcripts and higher transcript ratios) (Figure S2). Relative to the WT, the OE had 1.2-fold more pathogen-upregulated transcripts and a 1.2-fold higher transcript ratio associated with ‘UDP-glycosyltransferase activity’, and, respectively, 1.2-fold more pathogen-upregulated transcripts and a 1.1-fold higher transcript ratio associated with ‘glutathione transferase activity’ (Table S8), both previously associated with DON detoxification [19]. Thus, these results indicate a more pronounced induction of detoxification processes in the OE in response to *F. graminearum*, as compared to the WT.

### 2.8. Fusarium-Responsive KEGG Pathways Modulated by Overexpression of TaNACL-D1

**Figure 7:**
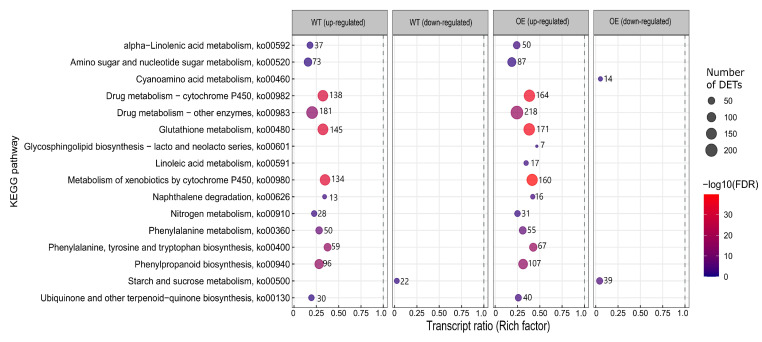
KEGG pathways significantly enriched in associated *Fusarium graminearum*-up/downregulated transcripts in the TaNACL-D1-overexpressing (OE) wheat cv. Fielder compared to the wild type (WT) at one day post-inoculation. The coloured scale on the right represents negative logarithmic scale with a base 10 of false discovery rate (FDR) values for each of these pathways. Abbreviation explanation: DETs, differentially expressed transcripts.

The OE and WT had, respectively, 20 and 18 KEGG pathways significantly enriched in pathogen-upregulated transcripts, and two and one KEGG pathways enriched in pathogen-downregulated transcripts. Figure 7 shows the 13 common KEGG pathways that were not equally enriched in OE compared to the WT as well as the three that were unique to the OE (none were unique to the WT). The 16 KEGG pathways common to both the OE and WT were more enriched in the OE as compared to the WT. These had >1.2-fold more pathogen-regulated genes and 1.2- to 2-fold higher transcript ratios associated with the OE as compared to the WT. The OE had, respectively, 1.4 more pathogen-upregulated transcripts and a 1.3-fold higher transcript ratio associated with the precursor of JA biosynthesis, ‘alpha-linolenic acid metabolism’, as compared to the WT (Figure S5). Fourteen of these transcripts were pathogen-upregulated only in the OE, while one was only upregulated in the WT. Thus, ‘alpha-linolenic acid metabolism’ was induced more often by *F. graminearum* in the OE as compared to the WT, based on both enrichment and level of expression. The induced transcripts code for enzymes involved in the synthesis of the volatile organic compound 3-hexenol (that suppressed the growth of *F. graminearum* in vitro [29] and JA (Figure S6). Another pathway, ‘linoleic acid metabolism’, was significantly enriched with pathogen-upregulated transcripts only in the OE (Figure 7). Transcripts coding for the enzyme linoleate 9S-lipoxygenase were pathogen-upregulated in the OE but not the WT (Figure S7) and this enzyme is involved in linoleic acid metabolism, oxylipin biosynthesis and defence against *Fusarium* [30,31]. Furthermore, the OE had 1.1-fold more pathogen-upregulated transcripts and a 1.1-fold higher transcript ratio associated with ‘phenylpropanoid biosynthesis’ and ‘phenylalanine metabolism’. This suggested a slightly more pronounced induction of phenylpropanoid biosynthesis-related transcripts in the OE, which is in line with results obtained by GO enrichment analysis (see Figures S2 and S4a, Table S4). Indeed, of the 94 pathogen-upregulated transcripts involved in ‘phenylpropanoid biosynthesis’ that were common to both the OE and WT, 89% of them had, on average, 2.5-fold higher expression in the OE than in the WT (Figure S8). These transcripts included those predicted to code for enzymes involved in lignin and coumarin biosynthesis, including cinnamyl alcohol dehydrogenase associated with *F. graminearum* resistance [32] (Figure S9). Two phenylpropanoid biosynthesis-associated transcripts were pathogen-upregulated only in the WT, and 13 were pathogen-upregulated only in the OE (Figure S8). Thus, based on the enrichment and level of expression, as compared to the WT the OE had more induced phenylpropanoid pathway activity.

‘Glycosphingolipid biosynthesis—lacto and neolacto series’ was enriched in pathogen-upregulated transcripts only in the OE (Figure 7) and glycosphingolipids and sphingolipids are known to be involved in pathogen defence and plant-programmed cell death (PCD) [33]. As stated earlier, detoxification processes play a role in defence against *F. graminearum* [19], and pathways related to detoxification (‘drug-related metabolism’, ‘glutathione metabolism’ and ‘metabolism of xenobiotics by cytochrome P450′) had 1.2-fold more associated pathogen-upregulated transcripts and a 1.2-fold higher transcript ratio in the OE compared to the WT, suggesting that the OE had more pronounced pathogen induction of detoxification-related processes as compared to the WT (Figure 7). ‘Cyanoamino acid metabolism’ has not been previously associated with *F. graminearum* defence but it was enriched in pathogen-downregulated transcripts only in the OE. The OE had a 1.8 higher number of pathogen-downregulated transcripts and a 2-fold higher transcript ratio associated with ‘starch and sucrose metabolism’, as compared to the WT (Figure 7), suggesting that primary metabolism was more compromised in the OE compared to the WT as a response to the pathogen.

### 2.9. F. graminearum-Dependent Transcripts under the Control of the TaNACL-D1 Transcription Factor

Thirteen transcripts were both pathogen-upregulated and expressed at a higher level in the OE as compared to the WT, while 23 were both pathogen-downregulated and expressed at a lower level in the OE as compared to the WT (Figure 2c). Thus, these 36 pathogen-responsive transcripts are potentially regulated by TaNACL-D1. Twenty-one of these transcripts were classified into seven gene ontology (GO) groups and had functionally characterised *Arabidopsis* and/or rice top BLAST homologs, while 15 did not (Tables S9 and S10, respectively). Of these 21 transcripts, five were associated with PCD, four with floral initiation, six with pollen development, three with embryo development and eight with biotic stress responses. They were grouped into five functional groups based on the characterised function of the Arabidopsis and rice homologs (Table 1). Functions indicated that TaNACL-D1 overexpression impacted development- and stress-related genes in response to *F. graminearum*. Of the eight associated with ‘biotic stress’, six coded for previously described papain-like cysteine proteases, a dicer protein and LOL1 isoform X2. Two additional downregulated transcripts coded for binding to TOMV RNA 1 (BTR1) [34] (‘transcription’ GO-group) and functionally characterised non-classified *T. aestivum* hessian fly response gene 1 protein (TaHfr1, [35]; not classified in GO-group). 

### 2.10. Genes Constitutively Regulated by TaNACL-D1

The 20 transcripts regulated by *TaNACL-D1* overexpression irrespective of the treatment and the time (i.e., at 0 dpi, 1-day post-mock and 1-day post-*Fusarium*) (Figure 2a) are the candidates most likely to be constitutively regulated by TaNACL-D1. These transcripts are listed in Table 2. Nine of these were upregulated and 11 downregulated due to TaNACL-D1 overexpression. Due to the lack of similarity in either their biological or molecular function, the transcripts were not grouped based on their gene ontology, and the function of 11 of the 20 genes was inferred based on that of their Arabidopsis or rice homologs (Tables S12 and S13). TaNACL-D1 and its homolog TaNACL-B1 were both constitutively upregulated due to TaNACL-D1 overexpression, and their Arabidopsis homolog was associated with xylem development [36]. Ubiquitin-like specific protease ESD4 (early in short days 4) isoform x1 was associated with floral initiation, flower development, embryo development and gametogenesis [20,37,38] and aspartic proteinase nepenthesin-1 was associated with primary and lateral root development [39]. Two of the 20 transcripts coded for up- and downregulated SHAGGY-like kinases associated with brassinosteroid signalling [40,41]. Two downregulated transcripts coded for proteins associated with a metabolic function: UDP (uridine diphosphate)-glucuronic acid decarboxylase 1 was associated with UDP-xylose biosynthesis [42], and phosphoglycerate kinase (cytosolic) was associated with glycolysis [43]. One upregulated transcript coded for the putative disease resistance RPP13 (Recognition of Peronospora parasitica 13) associated with pathogen defence [44]. Another downregulated transcript coded for auxin-induced protein 5NG4 associated with amino acid homeostasis [45] and one downregulated transcript coded for vacuolar cation/proton exchanger 2 isoform X2 associated with calcium ion transport [46]. These results indicate that TaNACL-D1 overexpression constitutively impacted genes involved in development, brassinosteroid signalling, transport, pathogen defence, and primary metabolism, irrespective of the treatment, and also regulated its chromosome B homolog.

## 3. Discussion

This is the first study to elucidate the impact of a NAC transcription factor on the wheat transcriptome. It provides insights into the biological impacts of an evolutionary divergent *NAC* gene on plant development and disease. Based on the fact that TaNACL-D1 reduced the spread of FHB symptoms on wheat heads and was activated as an early response to DON and FHB [18], herein the aim was to determine the impact of this gene on the transcriptome of wheat and its’ early response to *F. graminearum*. Almost two thirds of transcripts were pathogen-regulated both in the OE and WT and 95% of the biological processes that were pathogen-upregulated in the WT were also pathogen-upregulated in the TaNACL-D1-overexpressor (OE). This indicated that both shared highly similar defence response processes, which was expected given that the latter is a transgenic derivative of the former. Indeed, it is surprising that over a third of the transcripts were pathogen-regulated in a genotype-dependent, and thus potentially in a TaNACL-D1-associated, manner.

The enhanced transcriptional reprogramming in the OE as compared to the WT at 1 dpi as a response to the fungus may lead to a quicker defence response and hence less disease. A similar phenomenon was previously shown to occur during the early wheat response (30 hpi) to *F. graminearum*, with more pathogen-responsive genes detected in the FHB-resistant cv. CM-82036 as compared to four near isogenic lines (NILs) generated from a cross of the resistant cv. CM-82036 and susceptible cv. Remus [47]. In our study, more pathogen-induced transcriptional reprogramming in the OE compared to the WT manifested as greater enrichment in molecular functions such as ‘kinase activity’, ‘transcription regulator activity’ and ‘DNA-binding transcription factor activity’. More enhanced kinase expression was previously associated with FHB resistance [32,47]. Biological processes related to hormone pathways, detoxification, the phenylpropanoid pathway, oxidative stress and the immune response were more enriched in pathogen-upregulated transcripts in the OE, as compared to the WT. The main differences in the enrichment of pathogen-downregulated transcripts between the genotypes were in biological processes related to growth, cell cycle, DNA repair, cytoskeleton, and development. Many of these biological processes were also shown to be *Fusarium*-regulated in other studies [16,17,20]. Although analysis of the pathogen response to *TaNACL-D1* overexpression was not the focus of this study, it is interesting to note that there were slightly more fungal transcripts detected in the OE as compared to the WT. This warrants further investigation with more time points and may reflect an expanded pathogen response to the enhanced defence responses attributed to TaNACL-D1-overexpression.

The coordinated activation of hormones, starting with SA followed by JA, was proven to be important for enhanced FHB resistance [48]. In this study, the OE was more enriched in *Fusarium* upregulated transcripts associated with JA/SA/ET-related biological processes, as compared to the WT, concurring with findings of a previous transcriptome study comparing the pathogen response in FHB-resistant and susceptible cultivars [48]. The OE had more pronounced JA biosynthesis and JA-activated responses and signalling, as compared to the WT. The upregulation of JA biosynthesis-associated genes (‘methyl jasmonate esterase activity’ and ‘12−oxophytodienoate reductase activity’) in the OE compared to the WT suggests that TaNACL-D1 may impact the production of this hormone, acting either upstream of this pathway or through a feedback mechanism. The ‘biosynthesis of the oxylipins’, ‘alpha-linolenic acid metabolism’ and ‘linoleic acid metabolism’ were either more positively, or exclusively, enriched in the OE, as compared to the WT. Oxylipins are known to be important signalling molecules involved in pathogen defence [49]. Linoleic acid and alpha-linolenic acid are both precursors in the biosynthesis of various oxylipins, where alpha-linolenic acid is commonly known as a precursor of the oxylipin JA. Both acids are metabolised by linoleate-13S/9S-lipoxigenases (LOX13/LOX9) [49]. The OE was more enriched in pathogen-upregulated transcripts associated with ‘linoleate-13S-lipoxigenase activity’, and *LOX9* was pathogen-upregulated only in the OE. LOX9s were proven to be susceptibility factors in wheat and *Arabidopsis* during defence against *F. graminearum* infection, and knockdown of these *LOXs* in *Arabidopsis* led to attenuation of JA signalling and enhanced activation of SA signalling [50]. However, studies on maize LOX9s indicated contrasting effects of two different LOX9s on defence against *Fusarium verticillioides* [30,31]. Herein, the induction of LOX9 by *F. graminearum* in the OE may have facilitated defence against FHB, especially since JA-related biological processes and pathways were more positively enriched in the OE, as compared to the WT, but this warrants deeper investigation.

ABA and IAA have both been associated with susceptibility to FHB [16,20,21,51]. Herein, in response to the pathogen, the WT wheat (but not the OE) was positively enriched in transcripts associated with the ‘response to ABA’. ‘Positive regulation of the ‘ABA biosynthetic process’ was negatively impacted in the OE but not in the WT. These results suggest the ABA pathway was suppressed by the TaNACL-D1-mediated signalling pathway, potentially conferring higher resistance to the pathogen as compared to the WT. Regarding IAA, transcriptome results suggest that the pathogen-treated OE was slightly more enhanced than the WT in IAA biosynthesis, while the pathogen-treated WT but not the OE was positively enriched in the synthesis of IAA-AA. ‘Indole-containing metabolomic and biosynthetic processes’ and ‘methyl indole-3-acetate esterase activity’ were more positively enriched in the OE as compared to the WT response to *F. graminearum*. IAA-AAs are known as inactive forms of IAA intended for degradation or storage of IAA, except for IAA-Tryptophan (Trp) that inhibits IAA activity [52]. IAA-AAs have been associated with FHB susceptibility, being detected at much higher level in diseased spikelets of the susceptible cultivars compared to the more resistant ones [20]. Hence, enhanced IAA-AA synthesis in the WT versus OE may be associated with its increased susceptibility to FHB. Trp is the precursor of IAA biosynthesis [53] and ‘Trp metabolism’ was more positively enriched in pathogen-treated OE as compared to the WT. The enriched ‘Trp metabolism’ in the OE compared to the WT may have led to an increased carbon flux towards the synthesis of other indole-containing compounds, with the descendant ‘melatonin biosynthetic process’ being upregulated and enriched in response to the pathogen in the OE, but not the WT. Melatonin is a positive regulator of defence against fungal infection, reducing lesions, inhibiting fungal spread and damage caused by the infection, and preventing oxidative damage [54].

The hypersensitive response (HR) is one of the first lines of plant defence against pathogens, inducing the oxidative burst via the production of reactive oxygen species (ROS), which ultimately leads to programmed cell death (PCD) at the site of infection [55]. PCD is a favourable resistance mechanism against biotrophic pathogens that feed on live tissue. However, PCD favours the growth of necrotrophic pathogens that feed of dead tissue. Hemibiotrophs such as *F. graminearum* have evolved to trick the host by switching from the biotrophic to the necrotrophic phase, creating a complex task for the host to perfectly balance activation and deactivation of PCD [55]. The *TaNACL-D1*-overexpressing wheat may also be better able to defend itself against ROS, as compared to the WT, due to the exclusively or enhanced enrichment/activation of ‘hydrogen peroxide catabolic process’, ‘cellular oxidant detoxification’, ‘response to oxidative stress’, ‘oxygen binding’ and ‘oxygen carrier activity’. The OE may also negatively regulate the production of excess ROS by downregulating ‘photosystem I stabilization’ in response to *F. graminearum*. Photosystem I is a major site of ROS production [56]. Unlike the OE, the ‘phylloquinone biosynthetic process’ was enriched in WT pathogen-upregulated transcripts, and phylloquinones function as electron transporters in photosystem I [57]. It is therefore possible that the OE went through a more intense oxidative burst to inhibit the growth of the *F. graminearum* and, by one dpi, this genotype started to positively respond to the switch to the pathogens’ necrotrophic phase by having more enhanced antioxidant mechanisms to alleviate the oxidative burst and modulate PCD. It has been suggested that rapid induction of ROS and antioxidant enzymes increases resistance against FHB [28].

The OE was more enriched in pathogen-upregulated transcripts associated with detoxification and the phenylpropanoid-related processes and pathways, as compared to the WT. Detoxification processes are crucial for successful defence against DON which induces PCD to promote the switch to the necrotrophic phase of *F. graminearum* infection [11]. While effects were not statistically significant, there was a trend that showed a positive effect of *TaNACL-D1* overexpression on resistance to DON [18]. In response to *F. graminearum*, the OE was more enriched than in the WT in processes that could lead to trichothecene detoxification such as ‘xenobiotic transport’, ‘glutathione transferase activity’, ‘glutathione binding’ and ‘UDP glycosyltransferase activity’. UDP glycosyltransferase and glutathione transferase are associated with FHB resistance by forming inactivated DON conjugates [58,59,60,61]. Activation of the phenylpropanoid pathway and increased lignin content have been associated with FHB resistance and cell wall reinforcement, acting as a physical barrier that prevents fungal invasion and spread through the spike [17,25,26]. The results herein suggest that OE had more induced lignin biosynthesis than the WT, which could have contributed to an increased resistance to pathogen spread in the OE. Another *NAC* gene, *TaNAC032*, was recently shown to enhance resistance against FHB by regulating the production of lignin [9]. *TaNAC032* was localised in the defence-associated subfamily ‘a’, while *TaNACL-D1* was localised in the subfamily ‘h’ not associated with defence [15]. Thus, *TaNAC032* and *TaNACL-D1* are from divergent NAC subfamilies, but they may share a lignin-associated function in FHB resistance because of convergent evolution; this warrants further investigation.

Processes related to primary metabolism and development were pathogen-downregulated to a greater extent in the OE compared to the WT, possibly due to a reallocation of carbon sources towards secondary metabolic pathways and defence mechanisms. While jasmonates are known to be major inducers of defence mechanisms in plants, at the same time they are suppressors of growth-related hormones, cell cycle, cell proliferation, DNA biosynthesis, cell growth, photosynthesis and growth-related metabolites [62,63]. It is very likely that enhanced pathogen-induced JA-mediated defences in the OE led to down regulation of growth-related processes. It was reported that microtubules played a role in the accumulation of hydrogen peroxide and induction of PCD, which enhanced resistance against stripe rust disease in wheat [64]. Thus, downregulation of microtubule organization-related processes in the OE may be associated with the modulation of PCD in response to the *F. graminearum*. Suppression of PCD at the switch to the necrotrophic phase at one dpi could be favourable for the host and negative for the survival and spread of *F. graminearum*. Actin depolymerization was associated with stomatal closure in Arabidopsis [65] and stomatal closure is induced by JA [66]. Pathogen-downregulation of actin filament organization-related processes in the OE may be beneficial for stomatal defence, which is known to be regulated by NAC transcription factors [67], and this warrants further investigation. Stomatal closure was positively correlated with resistance to FHB in the resistant cv. Sumai3 at one dpi as compared to the susceptible cv. Rebelde [68].

In this study, 36 *F. graminearum* responsive transcripts were indirectly or directly both upregulated by TaNACL-D1 and primed by TaNACL-D1 to respond to the pathogen. PCD and defence against pathogens again emerge as TaNACL-D1-regulated processes when considering the function of many of these genes. Four putative papain-like cysteine protease (PLCP) peptidases were pathogen-downregulated only in the OE as a response to *TaNACL-D1* overexpression. Their common *Arabidopsis* homolog, CEP1 (Cysteine Endopeptidase 1), is involved in tapetal programmed cell death, pollen development, secondary cell wall thickening during xylem development, and in resistance to a biotrophic pathogen [69,70,71]. The top rice BLAST hit for these four cysteine proteases encodes proteinase SAG12-2 (senescence associated genes) which negatively regulated stress-induced cell death [72]. Another pathogen-downregulated gene in the OE but not in the WT is predicted to encode LOL1, and its’ *Arabidopsis* homolog is a positive regulator of PCD [73], while the rice homolog encodes a negative regulator of PCD [74]. Hence it is likely that downregulation of the four proteinases and LOL1 in the OE in response to FHB may be linked to modulation of PCD at one dpi; whether this leads to positive and/or negative regulation of PCD warrants further investigation. Arabidopsis LOL1 and SOD1 negatively and positively regulated accumulation of superoxide dismutase (SOD), respectively, consistent with their function in PCD control via the maintenance of ROS homeostasis [73]. A gene encoding a copper chaperone required for the activation of SOD was significantly pathogen-upregulated in the OE versus WT. Thus, pathogen-upregulation of the copper chaperone for SOD may be associated with the pathogen-downregulation of the putative wheat LOL1 in the TaNACL-D1 overexpressor.

Results in this study also suggest that TaNACL-D1 may regulate genes associated with developmental process: floral initiation, pollen germination and embryo development. In fact, only the OE was significantly enriched in *F. graminearum*-downregulated transcripts associated with ‘pollen development’, ‘positive regulation of embryonic development’ and ‘meristem maintenance’. Whether TaNACL-D1 regulates floral initiation/development-associated wheat genes as a response to the pathogen is debatable; genes involved in floral transition and initiation are expressed earlier during plant development and their expression depends on many environmental and endogenous cues [75]. However, some flower and embryo development associated transcripts were differentially expressed between genotypes at day zero, in the absence of pathogen treatment. One of the transcripts less abundant in the OE versus the WT encoded the MADS-box gene *TaSEP1-B5-2*, which is specifically expressed during inflorescence and seed development [76]. ‘TORC1 (Target of Rapamycin Complex I) signalling’ was pathogen-downregulated in the OE and TORC1 is a master regulator of signalling networks associated with environmental responses and development, and disruption of the complex components impairs flowering time and flower development [22]. Furthermore, TaNACL-D1 overexpression constitutively upregulated a ubiquitin-like-specific protease ESD4 (early in short days 4) isoform x1 associated with floral initiation, flower development, embryo development and gametogenesis [37,38]. The OE was enriched in pathogen-upregulated transcripts associated with processes that could lead to the biosynthesis of auxin, compared to the WT. Auxin plays a significant role in flower development [77]. Pathogen-induced modulation of indole-containing metabolic processes may be linked with altered developmental processes in the OE compared to the WT. 

Several interesting genes were constitutively regulated by TaNACL-D1, irrespective of the treatment. These included its chromosome B homolog. Given the low homology of TaNACL-D1 and TaNACL-B1 to characterized *Arabidopsis* NACs (<50% and the *Arabidopsis* hit belonged to a different (‘b’) subfamily, [15]), it is unlikely that they share a common function (xylem development). Another transcript constitutively regulated by TaNACL-D1 encodes an aspartic proteinase nepenthesin-1. Its’ Arabidopsis homolog was involved in root development [39] and, interestingly, preliminary results showed that TaNACL-D1 alters root growth under normal conditions when ectopically expressed in *Arabidopsis* [78]. Two transcripts that code for SHAGGY-like kinases were constitutively regulated (one up an one down) by TaNACL-D1 and their rice and *Arabidopsis* homologs are involved in brassinosteroid signalling [40,41]. One was highly TaNACL-D1-upregulated and the other one was downregulated. Brassinosteroids are important phytohormones regulating many developmental processes such as root, xylem, flower and stomatal development, and adaption to stress [79]. Thus, TaNACL-D1 may constitutively regulate developmental processes, particularly root development via regulation of the brassinosteroid pathway. Also, the role of the two GSK1 homologs may be divergent given their opposite expression profiles in response to TaNACL-D1 overexpression. These kinases may have undergone sub-functionalisation rather than the neo-functionalisation since they differ in one amino acid outside the conserved kinase domain.

## 4. Materials and Methods

### 4.1. Plant Material and Growth Conditions

*Triticum aestivum* (wheat) cultivar (cv) Fielder (the wild type, WT) and its *TaNACL-D1* (*Triticum aestivum NAC* like D1; TraesCS5D02G111300) overexpression derivative OE-2 described previously [18] were used in this research. Line OE-2 was chosen because it represented an average phenotype among the lines. The introduced TaNACL-D1 was driven by a rice *actin* promoter as described in detail by Perochon, Kahla et al. [18] Wheat cv. Fielder is susceptible to FHB disease [80], while the OE-2 line was more resistant to FHB [18]. Seeds were germinated in darkness for 96 h at 21 °C in 90 mm petri dishes on moist Whatman No. 1 filter paper (Whatman, UK). The germinated seedlings were transferred to 3 L pots containing John Innes compost No. 2 (Westland Horticulture, Dungannon, UK). All plants were grown under contained glasshouse conditions at 18 °C at night and 25 °C during the day with a 16 h light and a 8 h dark photoperiod at 300 µmol/m^2^/s and 70% relative humidity.

### 4.2. Fungal Material and Growth Conditions

*Fusarium graminearum* strain GZ3639 [81] used in the study was stored at −80 °C and, prior to use, was subcultured onto PDA (potato dextrose agar; Difco, Oxford, UK) plates and incubated at 25 °C for 5 days. Conidia were produced in Mung bean broth and adjusted to 2 × 10^6^ conidia/mL 0.02% Tween-20, as previously described [82].

### 4.3. FHB Experiment

Plants of cv. Fielder and OE-2 were grown as described above and the experiment comprised three independent trials. For FHB and mock-treated plants, at mid-anthesis (Zadoks growth stage 65; [83]), two florets of the two central spikelets were treated per plant with 10 μL of either 0.02% (*v/v*) Tween 20 (mock) or 2 × 10^6^ *Fusarium* conidia/mL 0.02% (*v/v*) Tween 20. Treated heads were covered with plastic bags to maintain high humidity and promote disease development (two days for disease assessment study and one day for RNA-seq analysis). For RNA-seq analysis, in each trial six heads from three individual plants (two from each plant) were harvested per genotype at (i) 0 h (non-treated), (ii) 1-day post-mock (Tween 20) treatment, and (iii) 1-day post *Fusarium*-treatment. Post-harvest, plant material was flash frozen in liquid nitrogen and stored at −70 °C prior to RNA extraction. For FHB disease assessment, at least 10 heads from mock and *Fusarium*-treated secondary tillers were scored per genotype. The level of infection was calculated by visually scoring the number of infected spikelets at 7, 14 and 21 days post-inoculation (dpi) and data were used to calculate area under the disease progress curve (AUDPC) [84].

### 4.4. RNA Extraction, cDNA Synthesis and qRT-PCR

Treated heads within a given experiment were pooled on a per-treatment basis prior to RNA extraction, resulting in 18 samples for RNA extraction (2 genotypes × 3 treatments/conditions × 3 independent trials). RNA was extracted from the spikelets using TRIzol™ (Invitrogen™) according to the manufacturer’s instructions. DNase treatment, quality control and cDNA synthesis were performed as described by Perochon, Kahla et al. [18]. The cDNA was used for qRT-PCR analysis to quantify *TaNACL-D1* transcript levels in OE-2 line compared to the WT treated with *F. graminearum* and Tween 20 (mock). *TaNACL-D1* transcript levels were quantified relative to both *YLS8 (*Yellow-leaf specific gene 8*;* TraesCS1D02G332500) and *TaPP2 (Triticum aestivum* Protein phosphatase 2A subunit A3; TraesCS5B02G165200*)* housekeeping genes, as described in Perochon, Kahla et al. [18].

### 4.5. RNA-Sequencing, Raw Count Statistical Analysis and DE Analysis

Paired-end sequencing of the libraries was performed by Beijing Genomics Institute (BGI, Shenzhen, China) using the BGISEQ-PE100 platform. Reads were filtered to remove adaptor sequences, contamination, and low-quality reads. The cDNA from bread wheat (*Triticum aestivum*) genome assembly version RefSeq v1.1 and *Fusarium graminearum* str. PH-1 were downloaded from the International Wheat Genome Sequencing Consortium (IWGSC; [85,86]) and Ensembl Fungi release 50, respectively. Filtered reads were mapped against the wheat and *F. graminearum* genomes in *Kallisto* [87]. For the Pearson correlation analysis and principle component analysis (PCA) of the expressed transcripts, mapped read counts were first normalised to transcript per million (tpm) using tximport [88]. The transcripts were deemed to be expressed if expression values of the given transcript were over 0.05 tpm in two out of the three trials. The Pearson correlation analysis between trials was done using Hmisc [89] and visualized using corrplot [90]. PCA for the 18 samples was conducted using ggplot2 [91]. Differential expression analysis, comparing genotypes (OE vs. WT) and treatments (*F. graminearum*, Tween 20 (mock)), was carried out using DeSeq2 on the *Kallisto* output abundance file [92]. Transcripts with an adjusted *p* value < 0.05 and that had a fold change value greater than two were deemed significantly differentially expressed. All scripts for this analysis were obtained from GitHub [93]. Genesis was used for visualization of differentially expressed transcripts (DETs) within a heat map [94].

### 4.6. Functional Annotation of F. graminearum-Regulated Transcripts

OmicsBox v1.4 [95] was used for functional annotation of *F. graminearum*-regulated transcripts at 1 dpi in the OE and the WT (all the following tools were accessed within the OmicsBox). All tools below were used with the default parameters. CloudBlast [95] was used to compare protein sequences encoded by a given transcript with a database of non-redundant protein sequences from *Viridiplantae*. The GO Mapping tool [95] was used to retrieve Gene ontology (GO) terms associated with BLASTP search hits. GO terms were then assigned to query protein sequences using the Blast2GO Annotation tool [95]. The CloudInterProScan (CloudIPS) tool [95] was used to classify query sequences into families, predict structural motifs/domains within the query sequences and assign them GO terms obtained through verified motifs/domains. InterProScan GOs results were added to the annotations based on the BLASTP results. The Enzyme Code Mapping tool [95] was used to assign enzyme codes to query sequences.

### 4.7. GO and KEGG Enrichment Analysis

GO and KEGG (Kyoto Encyclopaedia of Genes and Genomes) pathway enrichment analysis of wheat transcripts regulated by *F. graminearum* at 1 dpi in the OE and the WT was conducted using Fisher’s exact test in OmicsBox v1.4, using the False discovery rate (FDR) threshold of 0.05. Up- and downregulated transcripts were analysed separately for enrichment. The test set was a list of *F. graminearum*-regulated transcripts in the genotype (OE or WT), and the reference set was a set of all expressed transcripts detected by RNA-seq analysis in the corresponding genotype. For the purpose of studying hierarchically descent GO terms, the full list of enriched GO terms was reduced to hierarchically descent terms (hierarchically the lowest level) by separating them from the more general GO-terms (hierarchically higher level) post-Fisher’s exact test in OmicsBox v1.4.

### 4.8. Comparison of Regulated Transcripts with Arabidopsis and Rice Orthologs

The Blast Database tool in OmicsBox v1.4 [96] was used to create a database of peptide sequences from either *Arabidopsis* (*Arabidopsis thaliana*) (Araport11 genome release) or rice (*Oryza sativa*) proteomes (IRGSP v1.0 genome release). LocalBLAST tool (within OmicsBox) was used to compare query protein sequences encoded by *F. graminearum*- and/or TaNACL-D1-regulated transcripts with the aforementioned *Arabidopsis* and rice databases via BLASTP with default parameters (E-value cutoff 0.001).

### 4.9. Comparison of GO Terms and KEGG Pathways between Genotypes

Enriched GO terms and KEGG pathways responsive to *F. graminearum* at 1 dpi were compared between the genotypes by comparing their transcript counts (pathogen-regulated transcript count and reference-dataset-transcript counts compared between genotypes), and transcript ratios, also known as the Rich factors associated with the GO term/KEGG pathway (pathogen-regulated transcript count associated with the term/pathway relative to transcript count in the reference dataset). The higher the transcript ratio (Rich factor), the more enriched a GO term/pathway was in the *F. graminearum*-regulated transcripts [97]. The KEGG pathways and hierarchically descent GO-terms were compared between genotypes and were visualized in R using a previously described script [98]. GO terms with transcript ratio differences between genotypes >0.02, and transcript count differences compared between genotypes >1, were deemed to differ between genotypes; these cut-offs were arbitrarily chosen to focus on the GO terms that were most different between genotypes. All terms/pathways, where there was no difference in the pathogen-regulated transcript counts and the reference-dataset-transcript counts between genotypes, were deemed not to differ between the OE and WT.

### 4.10. Statistical Analysis

All statistical analysis was performed using the SPSS statistic software version 26 software for Windows 10. The normality of the data distribution was evaluated with the Shapiro–Wilk test. The Kruskal–Wallis test was used to compare differences between OE lines and WT for the mid-anthesis assessment; the Mann–Whitney test was used to compare the difference between OE-2 line and WT for the FHB disease assessment. The Kruskal–Wallis test was used to compare differences in TaNACL-D1 transcript levels between the OE-2 and WT.

## 5. Conclusions

In conclusion, this study highlighted that overexpression of the FHB resistance gene *TaNACL-D1* in wheat resulted in more pronounced transcriptional reprogramming as a response to fungal infection, potentially leading to enhanced defences such as detoxification, immune responses, secondary metabolism, hormone biosynthesis and signalling, etc., as determined via transcriptomic analysis. Results herein suggest that the regulation and response to JA and ABA are the primary hormone-mediated signalling pathways differentially regulated between the OE and the WT. Furthermore, results suggest that the OE may more efficiently regulate the oxidative burst and modulate PCD, thus putatively delaying the cell death necessary for the necrotrophic lifestyle of the pathogen. Delayed cell death coupled with a more pronounced induction of the phenylpropanoid pathway and lignin synthesis may explain the enhanced resistance to fungal spread associated with TaNACL-D1 overexpression, which warrants further investigation. The role, if any, of TaNACL-D1 in floral initiation and development also warrants further investigation. Future studies should focus on the functional characterisation of the TaNACL-D1 signalling module and determine the potential of this gene and its homologs as functional markers/GM targets for *Fusarium* resistance-breeding in wheat and other hosts of toxigenic *Fusarium* species.

## Figures and Tables

**Table 1:** List of putative candidate transcripts for enhanced resistance against *Fusarium graminearum* in TaNACL-D1 overexpressor.

Transcript ID ^1^	Gene Description ^2^	Functional Group	OE, *F.* *graminearum* Response (Fold Change)	*Fusarium*-Treatment Dependent Response to *TaNACL-D1* Overexpression (Fold Change)
Proteolysis				
TraesCS3D02G340500.1	Papain-like cysteine proteinase	Programmed cell death (PCD), biotic stress, pollen development	0.33	0.26
TraesCS3B02G378600.1	Papain-like cysteine proteinase	PCD, biotic stress, pollen development	0.32	0.43
TraesCS3D02G342100.1	Papain-like cysteine proteinase	PCD, biotic stress, pollen development	0.43	0.50
TraesCS6D02G089100.1	Aspartic proteinase Asp1-like	-	0.01	<0.01
TraesCS3B02G380400.1	Papain-like cysteine proteinase	PCD, biotic stress, pollen development	0.31	0.47
RNA-processing				
TraesCS3D02G411200.5	Endoribonuclease Dicer homolog 3a isoform X2	Floral initiation, biotic stress	0.11	0.23
TraesCS6B02G176700.1	Protein RRC1-like isoform X1	-	0.41	0.49
Transport				
TraesCS4B02G131700.2	Protein ZINC INDUCED FACILITATOR-LIKE 1 isoform X1	-	0.01	0.01
TraesCS3A02G323000.1	Protein MON2 homolog	-	0.08	0.03
TraesCS3A02G323000.2	Protein MON2 homolog	-	343.06	50.42
Transcription				
TraesCS2D02G294600.1	Transcription repressor OFP13	Floral initiation	5.25	6.39
TraesCS1A02G131000.2	Unnamed protein product	-	0.05	0.05
TraesCS6A02G109100.2 *	Protein arginine N-methyltransferase 5	Floral initiation	0.01	0.00
TraesCS5D02G383900.3	DNA-directed RNA polymerase II subunit RPB2	Embryo development	7.95	3.23
TraesCS1B02G431800.5	Protein BTR1	Biotic stress	0.01	0.01
Metabolic process				
TraesCS5D02G234000.1	ATP-dependent 6-phosphofructokinase 5, chloroplastic	-	0.02	0.01
TraesCS2B02G556100.3	DNA polymerase I A, chloroplastic-like	-	0.01	0.01
TraesCS2D02G396500.2	Copper chaperone for superoxide dismutase	-	213.55	167.07
TraesCS7B02G322500.2	4-amino-4-deoxychorismate synthase	-	0.01	0.01
TraesCS5D02G309600.1	Probable hydroxyacylglutathione hydrolase 2, chloroplastic	-	12.85	112.59
Developmental process				
TraesCS2B02G603800.1	Pentatricopeptide repeat-containing protein At5g04810, chloroplastic	Embryo development	0.02	0.02
TraesCS6A02G109100.2 *	Protein arginine N-methyltransferase 5	Floral initiation	0.01	<0.01
TraesCS3B02G367800.1	Phosphatidylinositol N-acetylglucosaminyltransferase subunit A isoform X1	Pollen development	0.01	0.01
Proteasomal degradation				
TraesCS5D02G112200.1	F-box/LRR-repeat protein 14-like	-	5.45	7.55
Other				
TraesCS4A02G151000.1	14-3-3-like protein GF14-D	-	2.75	2.89
TraesCS1B02G288600.2	Protein LOL1 isoform X2	PCD, biotic stress	0.01	0.01
TraesCS4B02G114700.1	Unnamed protein product	-	3.08	337.08
TraesCS7D02G103000.1	Very-long-chain 3-oxoacyl-CoA reductase 1-like	Embryo development	0.01	0.01
TraesCS5B02G271400.2	Unnamed protein product	-	2.53	3.41
TraesCS6A02G189100.3	40S ribosomal protein S5-1	-	0.19	0.16
TraesCSU02G004500.1	Uncharacterised protein LOC109739929	-	84.90	67.13
TraesCS1A02G272900.2	-	-	0.01	0.01
TraesCS2D02G176200.3	Predicted protein	-	0.01	0.02
TraesCS7D02G001300.1	Hessian fly response gene 1 protein	Biotic stress	0.27	0.19
TraesCS5B02G253400.2	F-box protein At3g07870-like isoform X1	-	0.01	0.01
TraesCS5B02G138000.1	Hypothetical protein TRIUR3_04453	-	0.01	0.01
TraesCS2D02G043300.1	Formin-like protein 5	Pollen development	2.53	7.83
TraesCS2B02G280900.1	Unnamed protein product	-	25.17	7.48
TraesCS1D02G383900.1	Predicted protein	-	107.96	85.31

^1^ Transcript marked with asterisk symbol (*) belongs to two GO-based groups. These transcripts were responsive to both *F. graminearum* and *TaNACL-D1* overexpression and were classified in GO-based group, and functional groups based on the homology to the functionally characterised top BLAST *Arabidopsis*/rice hit; Underlined transcripts had functionally characterised top BLAST *Arabidopsi*s and/or rice homolog and are listed in Tables S9 and S10. ^2^ The gene description is based on the description of the top *Viridiplantae* BLAST hit.

**Table 2:** List of putative transcripts constitutively modulated by TaNACL-D1 overexpression, irrespective of the treatment.

Transcript ID	Gene Description ^1^	Biological Process	Associated Function Based on Its Characterised *Arabidopsis*/Rice Top BLAST Hit ^2^	Response to *TaNACL-D1* Overexpression		
				No Treatment (Day Zero)- Dependent	Tween20 (Mock)- Dependent	*Fusarium-* Dependent
TraesCS5D02G111300.1	TaNACL-D1	Regulation of transcription, DNA-templated	Xylem development	48.5	33.0	17.9
TraesCS4B02G303500.2	GEM-like protein 1	-	-	3.7	6.5	6.1
TraesCS5A02G259000.1	SHAGGY-like kinase	Protein phosphorylation; negative regulation of brassinosteroid-mediatedsignalling pathway	Brassinosteroid signalling and salt tolerance	683.1	785.6	571.8
TraesCS1B02G005000.1	Ubiquitin-like-specific protease esd4 isoform x1	Proteolysis	Floral initiation, flower development, embryo development and gametogenesis, ABA signalling	2.5	3.1	3.3
TraesCS5B02G104200.1	TaNACL-B1	Regulation of transcription, DNA-templated	Xylem development	5.5	6.5	2.7
TraesCS2B02G242800.1	Unnamed protein product	-	-	6.4	11.3	8.6
TraesCS3A02G132200.1	Sec 20 family protein	Retrograde vesicle-mediated transport, Golgi to endoplasmic reticulum; membrane fusion	-	5.4	5.8	4.4
TraesCS4A02G469500.1	Aspartic proteinase nepenthesin-1	Metabolic process	Primary and lateral root development	2.1	2.3	2.5
TraesCS2B02G042700.2	Putative disease resistance RPP13- like protein 1	-	Pathogen defence	13.2	9.9	15.3
TraesCS2A02G237100.2	Nucleolar protein 58-like isoform X1	Ribosome biogenesis	-	<0.1	<0.1	<0.1
TraesCS1B02G093900.1	DNA-directed RNA polymerase III subunit RPC3	Transcription, DNA-templated	-	0.1	0.1	0.1
TraesCS3D02G349200.1	UDP-glucuronic acid decarboxylase 1	UDP-D-xylose biosynthetic process; D- xylose metabolic process	Biosynthesis of UDP-xylose	0.3	0.2	0.3
TraesCS6D02G174700.1	Protein phosphatase 2C and cyclic nucleotide- binding/kinase domain- containing protein isoform X1	Protein dephosphorylation; signal transduction; peptidyl-serine phosphorylation	-	0.2	0.2	0.2
TraesCS6A02G330100.1	Auxin-induced protein 5NG4	Transmembrane transport	Amino acid homeostasis in siliques	0.3	0.4	0.3
TraesCS4A02G156500.1	Vacuolar cation/proton exchanger 2 isoform X2	Cellular calcium ion homeostasis; calcium ion transmembrane transport	Calcium ion transport	0.4	0.3	0.3
TraesCS1B02G361800.1	SHAGGY-like kinase	Protein phosphorylation; negative regulation of brassinosteroid-mediated signalling pathway	Brassinosteroid signalling	0.4	0.4	0.4
TraesCS6B02G393800.1	Transmembrane 9 superfamily member 11	Protein localization to membrane	-	0.3	0.3	0.3
TraesCS7B02G375300.1	Phosphoglycerate kinase, cytosolic	Response to molecule of bacterial origin; gluconeogenesis; glycolytic process; protein phosphorylation; response to heat; response to light stimulus; response to glucose	Glycolysis	0.2	0.3	0.3
TraesCS2A02G237100.1	Nucleolar protein 58-like isoform X1	Ribosome biogenesis	-	<0.1	0.1	<0.1
TraesCSU02G061900.1	F-box protein At5g49610- like	Protein binding	-	0.1	0.1	0.1

^1^ The gene description is based on the description of the top *Viridiplantae* BLAST hit. ^2^ transcripts with functionally characterised *Arabidopsis* and/or rice top BLAST hit (hyphen those without).

## Data Availability

All data are available in the manuscript and the Supplementary material. The raw RNA-sequencing data, DESeq2 output data, GO and KEGG analysis output data associated with this study are openly available in FigShare: the raw RNA-sequencing data are available at https://doi.org/10.6084/m9.figshare.23540373.v1 (accessed on 21 June 2023).; the list of all expressed transcripts in each genotype x treatment combination is available at https://doi.org/10.6084/m9.figshare.23566125 (accessed on 21 June 2023); KEGG analysis data are available at https://doi.org/10.6084/m9.figshare.23540685.v2 (accessed on 21 June 2023); GO analysis data are available at https://doi.org/10.6084/m9.figshare.23540676.v2 (accessed on 21 June 2023); DESeq2 output data are available at https://doi.org/10.6084/m9.figshare.23540424.v2 (accessed on 21 June 2023).

## Supplementary Materials

The following supporting information can be downloaded at: https://www.mdpi.com/article/10.3390/plants12142708/s1, Table S1: Summary of the sequencing data generated for transcriptomes of wild type and TaNACL-D1 overexpressing cv. Fielder for given conditions after read filtering and genome mapping; Table S2: The total number of TaNACL-D1-regulated genes and the total number of corresponding TaNACL-D1-regulated transcripts in non-treated plants (day zero), Tween20 (mock)-treated plants at one day post-inoculation (dpi), and *Fusarium graminearum*-treated plants at 1 dpi; and the total number of *F. graminearum*-regulated transcripts and genes in the TaNACL-D1 overexpressing cv. Fielder or wild type; Table S3: total number of Gene ontology terms per three main gene ontology categories that were enriched in associated *Fusarium graminearum*-up/downregulated transcripts at one day post-inoculation in TaNACL-D1-overexpressing cv. Fielder and wild type; Table S4: Biological processes enriched in *Fusarium graminearum*-up/downregulated transcripts in the TaNACL-D1-overexpressing cv. Fielder and wild type at one day post-inoculation; Table S5: *Fusarium graminearum-*upregulated biological processes that are common to TaNACL-D1-overexpressing cv. Fielder and wild type at one day post-inoculation; Table S6: *Fusarium graminearum*-downregulated biological processes that are common to TaNACL-D1-overexpressing cv. Fielder and wild type at one day post-inoculation; Table S7: *Fusarium graminearum*-induced *Triticum aestivum* transcripts with putative function in cell-wall enforcement in the TaNACL-D1-overexpressing cv. Fielder as compared to the wild type [32,99]; Table S8: The most enriched 50 molecular functions associated with *Fusarium graminearum*-upregulated transcripts in the TaNACL-D1*-*overexpressing cv. Fielder and the wild type at one day post-inoculation; Table S9: The transcripts that were significantly *Fusarium*-regulated in the TaNACL-D1-overeexpressing cv. Fielder (OE) but not in the wild type (WT), and that were present at significantly higher or lower levels in the pathogen-treated OE versus WT, and that had functionally characterised *Arabidopsis thaliana* top BLAST hits [34,69,70,71,73,100,101,102,103,104,105,106,107,108,109,110,111,112,113,114,115,116,117,118,119,120]; Table S10: The transcripts that were significantly *Fusarium*-regulated in the *TaNACL-D1*-overeexpressing cv. Fielder (OE) but not in the wild type (WT), and that were present at significantly higher or lower levels in the pathogen-treated OE versus WT, and that had functionally characterised rice (*Oryza sativa*) top BLAST hits [72,121,122,123,124,125,126,127,128,129,130]; Table S11: The transcripts that were present at significantly higher or lower levels in the non-treated (day zero) TaNACL-D1*-*overexpressing cv. Fielder as compared to the wild type, and that were associated with development-related biological processes; Table S12: The transcripts that were present at significantly higher or lower levels in the TaNACL-D1-overexpressing cv. Fielder as compared to the wild type, irrespective of the treatment and that had functionally characterised Arabidopsis top BLAST hits [36,37,38,39,41,42,43,44,45,131,132]; Table S13: The transcripts that were present at significantly higher or lower levels in the *TaNACL-D1*-overexpressing cv. Fielder as compared to the wild type irrespective of the treatment and that had functionally characterised rice (*Oryza sativa*) top BLAST hits [40,46]; Figure S1: Quality assessment of RNA transcriptomes from 18 sequenced samples: (**a**) distribution of expressed transcripts across wheat subgenomes (A, B, D) and chromosomes (1–7), including an unknown subgenome/chromosome U; (**b**) number of expressed transcripts within each treatment x genotype combination; (**c**) the Pearson’s correlation of expressed transcripts between three trials. The correlation coefficient within each box represents each pairwise correlation; and (**d**) principal component (PC) plot of variance-stabilised-transformed read counts across 18 samples. Circles, triangles, and squares indicate *Fusarium graminearum* treatment, no treatment control (day zero) and Tween 20 treatment (mock), respectively. Colours refer to the genotypes: OE (overexpressing line) and WT (wild type); Figure S2: Descent biological processes associated with *Fusarium graminearum*-upregulated transcripts in the TaNACL-D1-overexpressing (OE) wheat cv. Fielder and the WT at one day post-inoculation. The coloured scale on the right represents a negative logarithmic scale with a base 10 of the false discovery rate (FDR) values for each of these processes. Abbreviation: DETs, differentially expressed transcripts; Figure S3: Descent biological processes associated with *Fusarium graminearum*-downregulated transcripts in the TaNACL-D1-overexpressing (OE) wheat cv. Fielder and the wild type (WT) at one day post-inoculation. The coloured scale on the right represents negative logarithmic scale with a base 10 of the False discovery rate (FDR) values for each of these processes. Abbreviation explanation: DETs = differentially expressed transcripts; Figure S4: Descent molecular functions significantly enriched with associated: (**a**) *Fusarium graminearum*-upregulated; and (**b**) *F. graminearum*-downregulated transcripts in the TaNACL-D1-overexpressing cv. Fielder (OE) and the wild type (WT). The coloured scale on the right represents negative logarithmic scale with a base 10 of the false discovery rate (FDR) values for each of these molecular functions. Abbreviations explanation: up = upregulated; down = downregulated; DETs = differentially expressed transcripts; Figure S5: The heatmap of the *F. graminearum*-upregulated transcripts associated with the alpha-linolenic acid metabolism at one day post-inoculation in the TaNACL-D1*-*overexpressing cv. Fielder (OE) and the wild type (WT). Enzyme codes: 1.1.1.1 = Alcohol dehydrogenase; 1.3.11.12 = linoleate 13S-lipoxygenase; 1.3.1.42 = 12-oxophytodienoate reductase; 2.3.1.16 = acetyl-CoA C-acyltransferase; 3.1.1.32 = phospholipase A1; 3.1.1.4 = phospholipase A2; 4.2.1.17 = enoyl-CoA hydratase; 4.2.1.92 = hydroperoxide dehydratase; 5.3.99.6 = allene-oxide cyclase. The coloured scale on the top represents log2 fold change. The white fields represent transcripts that are not regulated by the fungus. A dot within a coloured field indicates that the expression of the transcript in that genotype differed as compared to the other genotype. Abbreviation: DETs, differentially expressed transcripts; Figure S6: The map of the alpha-Linolenic acid metabolism KEGG pathway (map00592) [133]. The coloured enzyme codes are associated with the *F. graminearum*-upregulated transcripts at one day post-inoculation in both the *TaNACL-D1*-overexpressing cv. Fielder and the wild type. Enzyme codes: 1.1.1.1 = alcohol dehydrogenase1.3.11.12 = linoleate 13S-lipoxygenase;.1.3.1.42 = 12-oxophytodienoate reductase; 2.3.1.16 = acetyl-CoA C-acyltransferase; 3.1.1.32 = phospholipase A1; 3.1.1.4 = phospholipase A2; 4.2.1.17 = enoyl-CoA hydratase; 4.2.1.92 = hydroperoxide dehydratase; 5.3.99.6 = allene-oxide cyclase; Figure S7: The map of the linoleic acid metabolism KEGG pathway (map00591) [133]. The coloured enzyme codes are associated with the *F. graminearum*-upregulated transcripts at one day post-inoculation in only the *TaNACL-D1-*overexpressing cv. Fielder (OE) or in both the OE and the wild type (OE/WT) (highlighted with blue font). Enzyme codes: 1.3.11.12 = linoleate 13S-lipoxygenase; 1.3.11.58 = linoleate 9S-lipoxygenase; 3.1.14 =phospholipase A(2); Figure S8: The heatmap of the *F. graminearum*-upregulated transcripts associated with the phenylpropanoid biosynthesis at one day post-inoculation in the *TaNACL-D1-*overexpressing cv. Fielder (OE) and the wild type (WT). Enzyme codes: 1.1.1.195 = cinnamyl alcohol dehydrogenase; 1.4.14.91 = Trans-cinnamate 4-monooxygenase; 1.2.1.44 = Cinnamoyl-CoA reductase; 3.2.1.21 = beta-glucosidase; 4.3.1.24/25 = phenylalanine/tyrosine ammonia-lyase; 6.2.1.12 = 4-coumarate—CoA ligase. The coloured scale on the top represents log2 fold change. The white fields represent transcripts that are not regulated by the fungus. A dot within a coloured field indicates that the expression of the transcript in that genotype differed as compared to the other genotype. Abbreviation explanation: DETs; differentially expressed transcripts; Figure S9: The map of the phenylpropanoid biosynthesis KEGG pathway (map00940) [133]. The coloured enzyme codes are associated with the *F. graminearum*-upregulated transcripts associated with phenylpropanoid biosynthesis at one day post-inoculation in the *TaNACL-D1-*overexpressing cv. Fielder (OE) and in the wild type (WT). Enzyme codes explanation: 1.1.1.195 = cinnamyl alcohol dehydrogenase; 1.4.14.91 = trans-cinnamate 4-monooxygenase; 1.2.1.44 = C=cinnamoyl-CoA reductase; 3.2.1.21 = beta-glucosidase; 4.3.1.24/25 = phenylalanine/tyrosine ammonia-lyase; 6.2.1.12 = 4-coumarate--CoA ligase.
