# Supplementary material for: Transcriptional Profiling Reveals the Wheat Defences against Fusarium Head Blight Disease Regulated by a NAC Transcription Factor
Source: Plants (Basel). 2023 Jul 20;12(14):2708. doi: 10.3390/plants12142708 (PMC10383764; doi:10.3390/plants12142708)
Supplement: Supplementary file 1 [file plants-12-02708-s001.zip › plants-2285691-supplementary.pdf]

Supplemental Tables and Figures

Table S1 Summary of the sequencing data generated for transcriptomes of wild type and TaNACL-D1-overexpressing cv. Fielder for given conditions after read filtering and genome mapping.

Condition/sample[1]	Clean reads	Total mapped (%)[2]	Uniquely mapped (%)[3]	Q20(%)
WT_*Fg*_rep1_1dpi	49443988	83.9	55.6	96
WT_*Fg*_rep2_1dpi	49526014	86.2	56.2	96
WT_*Fg*_rep3_1dpi	49585466	86.5	56.9	96
OE_*Fg*_rep1_1dpi	48477590	87	59.8	96
OE_*Fg*_rep2_1dpi	49496904	86.6	57.2	96
OE_*Fg*_rep3_1dpi	49742842	86.6	57.2	96
WT_Tween20_rep1_1dpi	49969834	85.1	52.7	96
WT_Tween20_rep2_1dpi	48481434	85.5	53.5	97
WT_Tween20_rep3_1dpi	48822502	85.4	53.5	97
OE_Tween20_rep1_1dpi	48335298	85.3	53.3	97
OE_Tween20_rep2_1dpi	50087594	85.2	53.3	97
OE_Tween20_rep3_1dpi	50185508	85.5	53.3	97
WT_rep1_0dpi_NT	48444492	85.4	53.1	97
WT_rep2_0dpi_NT	48232922	85.5	52.7	97
WT_rep3_0dpi_NT	48449112	85.4	52.3	97
OE_rep1_0dpi_NT	50045512	85.6	52.7	96
OE_rep2_0dpi_NT	48691474	85.9	53.5	97
OE_rep3_0dpi_NT	48585640	85.4	52.8	97

[1] WT= Wild type; OE = *TaNACL-D1* overexpressor; *Fg* = *Fusarium graminearum* treatment; Tween20 = mock treatment; NT = no treatment; dpi = days post-inoculation; Rep1-3 = replicates 1-3. [2]Percentage of fragments that could be mapped to target sequences out of the total number of processed paired-end clean reads. [3]Percentage of fragments that could be mapped to a unique target sequence out of the total number of processed paired-end clean reads.

Table S2 The total number of TaNACL-D1-regulated genes and the total number of corresponding TaNACL-D1-regulated transcripts in non-treated plants (day zero), Tween20 (mock)-treated plants at one day post-inoculation (dpi), and *Fusarium graminearum*-treated plants at one dpi; and the total number of *F. graminearum*-regulated transcripts and genes in the TaNACL-D1-overexpressing cv. Fielder or wild type.

Comparison[1]	Nr of DEGs[2]	Nr of DETs[3]
NT_0dpi_OEvsWT	264	272
Tween20_1dpi_OEvsWT	167	172
*Fg*_1dpi_OEvsWT	140	147
WT_1dpi_*Fg*vsTween20	6452	6704
OE_1dpi_*Fg*vsTween20	8322	8704

[1] WT= Wild type; OE = *TaNACL-D1* overexpressor; *Fg* = *Fusarium graminearum* treatment; Tween20 = mock treatment; NT = no treatment; dpi = days post inoculation; vs = versus. [2] Nr= number; DEGs= differentially expressed genes. [3] DETs = differentially expressed transcripts.

Table S3 Total number of gene ontology terms per three main gene ontology categories that were enriched in associated *Fusarium graminearum*-up/downregulated transcripts at one day post-inoculation in TaNACL-D1-overexpressing cv. Fielder and wild type.

GO category	Number of upregulated GO-terms[1]		Number of downregulated GO-terms[1]	
	OE[1]	WT[1]	OE[1]	WT[1]
Biological process	358	315	169	36
Molecular function	232	218	77	42
Cellular compartment	23	20	32	4

[1] GO= Gene ontology; OE = *TaNACL-D1*-overexpressing cv. Fielder; WT = wild type.

Table S4 Biological processes enriched in *Fusarium graminearum*-up/downregulated transcripts in the TaNACL-D1-overexpressing cv. Fielder and wild type at one day post-inoculation.

	OE[1]				WT[1]			
Biological process[2]	FDR[3]	Number of *F. graminearum*-regulated transcripts	Number of transcripts in the reference[4]	Transcript ratio[5]	FDR[3]	Number of *F. graminearum*-regulated transcripts	Number of transcripts in the reference[4]	Transcript ratio[5]
**ABSCISIC ACID**								
Response to abscisic acid	-	-	-	-	0.036748995	70	596	0.1
Positive regulation of abscisic acid biosynthetic process	0.002189114	3	4	0.8	-	-	-	-
**SALICYLIC ACID**								
Cellular response to salicylic acid stimulus	3.1E-05	22	69	0.3	0.000253	18	69	0.3
Positive regulation of salicylic acid mediated signalling pathway	0.002543	4	4	1	0.035366	3	4	0.8
Regulation of salicylic acid biosynthetic process	2.18E-06	17	38	0.4	4.62E-05	13	34	0.4
Regulation of salicylic acid mediated signalling pathway	3.88E-05	18	50	0.4	0.001131	14	51	0.3
Regulation of salicylic acid metabolic process	2.67E-07	21	50	0.4	7.56E-05	15	46	0.3
Response to salicylic acid	2.59E-12	48	142	0.3	2.39E-09	37	135	0.3
Salicylic acid biosynthetic process	5.19E-06	17	40	0.4	9.46E-05	13	36	0.4

Table S4 continued…

Biological process[2]	OE[1] FDR[3]	Number of *F. graminearum*-regulated transcripts	Number of transcripts in the reference[4]	Transcript ratio[5]	WT[1] FDR[3]	Number of *F. graminearum*-regulated transcripts	Number of transcripts in the reference[4]	Transcript ratio[5]
Salicylic acid mediated signalling pathway	1.74E-05	20	57	0.4	0.00035	16	58	0.3
Salicylic acid metabolic process	1.16E-15	41	90	0.5	8.53E-13	33	86	0.4
**JASMONIC ACID**								
Response to jasmonic acid	1.28E-19	67	180	0.4	3.49E-11	46	172	0.3
Jasmonic acid mediated signalling pathway	6.06E-10	40	121	0.3	0.000259	25	117	0.2
Cellular response to jasmonic acid stimulus	5.28E-09	40	129	0.3	0.000658	25	124	0.2
Regulation of jasmonic acid mediated signalling pathway	2.25E-13	33	69	0.5	5.26E-08	23	66	0.3
Jasmonic acid metabolic process	7.06E-09	29	76	0.4	3.57E-06	22	75	0.3
Jasmonic acid biosynthetic process	0.024225	9	28	0.3	-	-	-	-
**GIBBERELLIC ACID**								
Gibberellic acid mediated signalling pathway	9.9E-06	24	75	0.3	0.00023	19	75	0.3
Negative regulation of gibberellic acid mediated signalling pathway	1.54E-16	22	26	0.8	4.59E-14	18	23	0.8
Regulation of gibberellic acid mediated signalling pathway	1.01E-08	22	47	0.5	3.51E-07	18	46	0.4
**ETHYLENE**								
Response to ethylene	7.67E-19	80	249	0.3	1.89E-12	58	235	0.2
Ethylene-activated signalling pathway	1.43E-12	54	170	0.3	9.89E-08	38	160	0.2

Table S4 continued…

Biological process[2]	OE[1] FDR[3]	Number of *F. graminearum*-regulated transcripts	Number of transcripts in the reference[4]	Transcript ratio[5]	WT[1] FDR[3]	Number of *F. graminearum*-regulated transcripts	Number of transcripts in the reference[4]	Transcript ratio[5]
Cellular response to ethylene stimulus	5.18E-12	54	175	0.3	1.99E-07	38	164	0.2
**INDOLE-ACETIC ACID**								
Indole-containing compound metabolic process	1.57E-22	60	133	0.5	4.84E-21	52	130	0.4
Indole-containing compound biosynthetic process	8.16E-25	56	108	0.5	6.56E-23	49	107	0.5
**PHENYLPROPANOID PATHWAY**								
Phenylpropanoid metabolic process	1.56E-41	107	231	0.5	1.18E-40	96	230	0.4
Phenylpropanoid biosynthetic process	1.13E-34	79	154	0.5	1.54E-32	70	154	0.5
**DETOXIFICATION**								
Cellular detoxification	3.82E-13	119	548	0.2	1.96E-14	106	550	0.2
Cellular oxidant detoxification	1.95E-11	109	513	0.2	2E-13	99	515	0.2
Cellular detoxification of aldehyde	0.014544	10	31	0.3	-	-	-	-

[1] OE = *TaNACL-D1*-overexpressing cv. Fielder; WT = wild type.

[2] Underlined biological process is downregulated.

[3] FDR = False discovery rate (Fischer's exact test, FDR <0.05).

[4] Reference = The number of all expressed transcripts associated with the biological process in the corresponding genotype.

[5] Transcript ratio = The number of *F. graminearum*-regulated transcripts associated with the biological process relative to the number of transcripts associated with the same biological process in the reference (the number of all expressed transcripts associated with the biological process in the corresponding genotype).

Table S5 *Fusarium graminearum*-upregulated biological processes that are common to TaNACL-D1-overexpressing cv. Fielder and wild type at one day post-inoculation.

Biological processes	Number of transcripts in the reference (OE) [1,2]	Transcript ratio (OE) [1,3]	Number of transcripts in the reference (WT) [1,2]	Transcript ratio (WT) [1,3]	Ratio of Transcript ratios in OE compared to WT [1,4]
Carboxylic acid metabolic process	2662	0.2	2656	0.1	1.2
Cell communication	3015	0.2	3008	0.1	1.2
Cellular protein modification process	8586	0.1	8582	0.1	1.3
Cellular response to chemical stimulus	2013	0.2	2004	0.1	1.2
Cellular response to stimulus	4868	0.1	4864	0.1	1.2
Defence response	2020	0.2	2000	0.2	1.2
Defence response to other organism	962	0.3	955	0.2	1.2
Interspecies interaction	962	0.3	1120	0.2	1.3
Immune response	962	0.3	955	0.2	1.2
Immune system process	1132	0.3	955	0.2	1.1
Macromolecule modification	9305	0.1	9304	0.1	1.3
Organic acid biosynthetic process	1316	0.2	1323	0.2	1.2
Organic acid metabolic process	2771	0.2	2766	0.1	1.2
Organonitrogen compound metabolic process	16126	0.1	16128	0.1	1.2
Oxoacid metabolic process	2694	0.2	2687	0.1	1.2
Phosphate-containing compound metabolic process	7455	0.1	7475	0.1	1.2
Phosphorus metabolic process	7551	0.1	7575	0.1	1.2
Phosphorylation	5198	0.2	5227	0.1	1.2
Protein modification process	8586	0.1	8582	0.1	1.3
Protein phosphorylation	3969	0.2	3972	0.2	1.2
Response to biotic stimulus	1160	0.3	1151	0.3	1.2
Response to chemical	3306	0.2	3284	0.1	1.2
Response to endogenous stimulus	1752	0.2	1724	0.1	1.3
Response to external biotic stimulus	1065	0.3	1055	0.2	1.2
Response to external stimulus	1425	0.2	1415	0.2	1.2
Response to hormone	1733	0.2	1705	0.1	1.3
Response to organic substance	2210	0.2	2185	0.1	1.3
Response to other organism	1065	0.3	1055	0.2	1.2
Response to stimulus	8495	0.1	8476	0.1	1.2
Response to stress	4996	0.1	4968	0.1	1.2
Signal transduction	2675	0.2	2677	0.1	1.2
Signalling	2680	0.2	2682	0.1	1.2
Small molecule biosynthetic process	1826	0.2	1836	0.1	1.2
Small molecule metabolic process	4484	0.1	4484	0.1	1.2

Table S5 continued…

Biological processes	Number of transcripts in the reference (OE) [1,2]	Transcript ratio (OE) [1,3]	Number of transcripts in the reference (WT) [1,2]	Transcript ratio (WT) [1,3]	Ratio of Transcript ratios in OE compared to WT [1,4]
Small molecule metabolic process	4484	0.1	4484	0.1	1.2
Transmembrane transport	3458	0.1	3446	0.1	1.2

[1] OE = *TaNACL-D1*-overexpressing cv. Fielder; WT = wild type.

[2] Reference = All expressed transcripts in the corresponding genotype.

[3] Transcript ratio = The number of *F. graminearum*-regulated transcripts associated with the biological process relative to the number of transcripts associated with the same biological process in the reference (all expressed transcripts in the corresponding genotype).

[4] Ratio of the transcript ratios = The transcript ratio values compared between genotypes by dividing the value from the OE with the value from the WT.

Table S6 *Fusarium graminearum*-downregulated biological processes that are common to TaNACL-D1-overexpressing cv. Fielder and wild type at one day post-inoculation.

Biological processes	Number of transcripts in the reference (OE) [1,2]	Transcript ratio (OE) [1,3]	Number of transcripts in the reference (WT) [1,2]	Transcript ratio (WT) [1,3]	Ratio of Transcript ratios in OE compared to WT [1,4]
(1->3)-beta-D-glucan biosynthetic process	44	0.16	45	0.11	1.43
Actin cytoskeleton organization	288	0.10	281	0.05	2.25
Actin filament organization	271	0.10	264	0.05	2.10
Actin filament-based movement	48	0.17	45	0.13	1.25
Actin filament-based process	291	0.10	284	0.05	2.25
Actin filament-based transport	48	0.17	45	0.13	1.25
Anatomical structure morphogenesis	740	0.06	750	0.03	1.75
Cell morphogenesis	305	0.08	314	0.05	1.61
Cell plate assembly	20	0.30	20	0.25	1.20
Chromosome organization	1746	0.05	1768	0.02	1.84
Cytoskeleton-dependent intracellular transport	61	0.13	57	0.11	1.25
Demethylation	95	0.12	99	0.08	1.43
DNA demethylation	39	0.13	39	0.13	1.00
Establishment of localization in cell	2208	0.04	2202	0.02	1.53
Establishment of vesicle localization	106	0.09	105	0.08	1.24
Intracellular transport	2131	0.04	2123	0.02	1.50
Movement of cell or subcellular component	252	0.09	244	0.05	2.02
Nuclear transport	386	0.07	380	0.05	1.48
Nucleocytoplasmic transport	386	0.07	380	0.05	1.48
Protein import into nucleus	172	0.08	169	0.05	1.42
Regulation of anatomical structure morphogenesis	146	0.12	148	0.06	2.03
Regulation of autophagy	29	0.21	27	0.15	1.40
Regulation of cell morphogenesis	106	0.15	109	0.08	1.83
Regulation of cell shape	89	0.18	90	0.10	1.80
Vesicle cytoskeletal trafficking	48	0.17	45	0.13	1.25
Vesicle localization	106	0.09	105	0.08	1.24
Vesicle transport along actin filament	48	0.17	45	0.13	1.60

[1] OE = *TaNACL-D1*-overexpressing cv. Fielder; WT = wild type.

[2] Reference = All expressed transcripts in the corresponding genotype.

[3] Transcript ratio = The number of *F. graminearum*-regulated transcripts associated with the biological process relative to the number of transcripts associated with the same biological process in the reference (all expressed transcripts in the corresponding genotype).

[4] Ratio of the transcript ratios = The transcript ratio values compared between genotypes by dividing the value from the OE with the value from the WT.

Table S7 *Fusarium graminearum*-induced *Triticum aestivum* transcripts with putative function in cell-wall enforcement in the *TaNACL-D1*-overexpressing cv. Fielder as compared to the wild type.

Transcript ID[1]	Description based on the top *Viridiplantae* BLAST hit	Wild type, *F. graminearum* response		*TaNACL-D1*-overexpressor, *F. graminearum* response	
		Fold change[2]	False discovery rate	Fold change[2]	False discovery rate
TraesCS2A02G490000.1	Agmatine coumaroyltransferase	26.2	0.002016799	**136.6**	3.09351E-05
TraesCS2A02G490700.1	Agmatine coumaroyltransferase	**402.1**	3.20747E-21	383.9	6.77792E-44
TraesCS2B02G518300.1	Agmatine coumaroyltransferase	13.3	5.5981E-07	**15.7**	2.0043E-05
TraesCS2B02G518900.1	Agmatine coumaroyltransferase	33.8	5.94079E-24	**48.6**	2.76263E-69
TraesCS2B02G519100.1	Agmatine coumaroyltransferase	17.8	2.42961E-22	**26.4**	2.98783E-56
TraesCS2D02G490900.1	Agmatine coumaroyltransferase	91.3	2.06536E-06	**104.1**	1.22003E-11
TraesCS2D02G491100.1	Agmatine coumaroyltransferase	80.0	4.02995E-42	**118.8**	3.3154E-99
TraesCS1A02G088300.1	Tryptamine hydroxycinnamoyltransferase 2-like	63.8	7.47387E-08	**79.4**	1.75298E-19
TraesCS1B02G107300.1	Tryptamine hydroxycinnamoyltransferase 2-like	**194.9**	1.79332E-05	148.3	3.25294E-07
TraesCS1B02G107400.1	Tryptamine hydroxycinnamoyltransferase 2-like	**8.8**	0.000328716	8.7	7.4906E-08
TraesCS1B02G107500.1	Tryptamine hydroxycinnamoyltransferase 2-like	13.0	0.000122439	**14.4**	1.13826E-09
TraesCS1D02G089900.1	Tryptamine hydroxycinnamoyltransferase 2-like	3.8	0.007365152	**4.6**	1.52848E-10
TraesCS2A02G354500.1	Hydroxycinnamoyltransferase 1	2.1	7.9669E-10	**2.4**	1.17209E-09
TraesCS2D02G354300.1	Hydroxycinnamoyltransferase 1	-	-	2.6	8.20196E-12
TraesCS5A02G337500.1	Putrescine hydroxycinnamoyltransferase 3-like	210.3	1.32217E-30	**644.1**	8.01383E-39
TraesCS5A02G337700.1	Putrescine hydroxycinnamoyltransferase 3-like	149.0	4.12867E-06	**199.8**	7.7686E-08
TraesCS5A02G337800.1	Putrescine hydroxycinnamoyltransferase 3-like	83.7	2.89268E-07	**362.5**	3.2175E-10
TraesCS5A02G337900.1	Putrescine hydroxycinnamoyltransferase 3-like	38.7	0.000330941	**220.7**	4.19648E-08
TraesCS5A02G338600.1	putrescine hydroxycinnamoyltransferase-like	14.0	4.21843E-10	**25.2**	6.19772E-12
TraesCS5B02G304300.1	Putrescine hydroxycinnamoyltransferase 3-like	**69.2**	1.1235E-10	55.2	2.06221E-17
TraesCS5B02G336300.1	Putrescine hydroxycinnamoyltransferase 3-like	118.5	1.83358E-20	118.5	8.53327E-38
TraesCS5B02G336500.1	Putrescine hydroxycinnamoyltransferase 3-like	119.7	2.15903E-06	**334.7**	6.90041E-10
TraesCS5B02G336600.1	Putrescine hydroxycinnamoyltransferase 3-like	**312.9**	1.57513E-08	130.5	1.49004E-09

Table S7 continued…

Transcript ID[1]	Description based on the top *Viridiplantae* BLAST hit	Wild type, *F. graminearum* response		*TaNACL-D1*-overexpressor, *F. graminearum* response	
		Fold change[2]	False discovery rate	Fold change[2]	False discovery rate
TraesCS5B02G336700.1	Putrescine hydroxycinnamoyltransferase 3-like	12.9	1.2434E-07	**18.1**	3.87003E-11
TraesCS5B02G337000.1	Putrescine hydroxycinnamoyltransferase-like	19.2	9.60135E-07	**29.2**	1.12996E-08
TraesCS5D02G310800.1	Putrescine hydroxycinnamoyltransferase 3-like	-	-	67.9	0.017339424
TraesCS5D02G342100.1	Putrescine hydroxycinnamoyltransferase 3-like	130.1	8.33446E-40	**160.2**	9.99816E-85
TraesCS5D02G342300.1	Putrescine hydroxycinnamoyltransferase 3-like	**261.3**	5.24071E-07	68.0	5.00151E-09
TraesCS5D02G342400.1	Putrescine hydroxycinnamoyltransferase 3-like`	`	\	**194.7**	3.37646E-07
TraesCS5D02G342700.1	Putrescine hydroxycinnamoyltransferase-like	23.7	4.29895E-18	**32.8**	1.45818E-21
TraesCS5D02G342800.1	Putrescine hydroxycinnamoyltransferase-like	**7.7**	0.001121899	5.2	0.00419115
TraesCS6A02G232200.1	Hydroxycinnamoyltransferase 2	2.3	2.70082E-06	**3.4**	3.16721E-18
TraesCS6D02G203000.1	Hydroxycinnamoyltransferase 2	2.3	1.09564E-06	**3.0**	2.0977E-20
TraesCS7B02G054100.1	Hydroxycinnamoyltransferase 4-like	11.1	8.95283E-06	**18.7**	9.62438E-19
TraesCS5D02G488800.2	Caffeic acid O-methyltransferase	**1441.4**	1.33006E-13	981.7	1.65969E-13
TraesCS5D02G488900.1	Caffeic acid O-methyltransferase	**2195.4**	8.55349E-16	951.1	1.04936E-13
TraesCS7A02G333900.1	Caffeic acid O-methyltransferase	2.1	5.79495E-07	**2.5**	7.92317E-19
TraesCS7B02G245500.1	Caffeic acid O-methyltransferase	2.4	9.23752E-09	**3.1**	1.3994E-30
TraesCS7D02G341500.1	Caffeic acid O-methyltransferase	2.4	3.07016E-08	**3.2**	1.19716E-47
TraesCS7A02G127600.1	Caffeoyl-CoA O-methyltransferase 1	6.6	6.98663E-25	**10.3**	2.6911E-125
TraesCS7B02G027200.1	Caffeoyl-CoA O-methyltransferase 1	4.4	5.35854E-16	**5.7**	2.81754E-62
TraesCS7B02G136100.1	Caffeoyl-CoA O-methyltransferase 2	18.6	1.01761E-13	**21.4**	7.63642E-27
TraesCS7D02G126000.1	Caffeoyl-CoA O-methyltransferase 1	4.4	2.14511E-17	**6.6**	2.57338E-45
TraesCS2D02G489700.1	WRKY45-like transcription factor	3.8	2.87185E-08	**5.9**	1.13067E-27

[1] TraesCS5B02G336600 (Traes_5AL_E23B0E6C4) and TraesCS5D02G488900 (Traes_5DL_7F0CD0F79) were *F. graminearum*-induced only in resistant recombinant inbred line carrying resistant allele of *QTL-Fhb2* in [32]; TraesCS2D02G489700.1 codes for TaWRKY70 [99].

[2] Values that are higher in one genotype compared to the other are highlighted in bold.

Table S1 The most enriched 50 molecular functions associated with *Fusarium graminearum*-upregulated transcripts in the TaNACL-D1-overexpressing cv. Fielder and the wild type at one day post-inoculation.

	WT[1]				OE[1]			
Molecular function[2]	FDR [3]	Number of *F. graminearum*-regulated transcripts	Number of transcripts in the reference[4]	Transcript ratio[5]	FDR [3]	Number of *F. graminearum*-regulated transcripts	Number of transcripts in the reference[4]	Transcript ratio[5]
Catalytic activity	1.18371E-88	2985	27108	0.11	1.08078E-91	3648	27130	0.13
Ion binding	1.12614E-58	1972	17281	0.11	4.61019E-54	2369	17301	0.14
Organic cyclic compound binding	0.001321893	1845	20649	0.09	0.00069574	2298	20624	0.11
Heterocyclic compound binding	0.001199349	1843	20615	0.09	0.000527387	2297	20591	0.11
Transferase activity	5.61145E-58	1476	12058	0.12	5.17446E-67	1818	12052	0.15
Small molecule binding	2.58302E-35	1228	10585	0.12	3.02048E-34	1480	10588	0.14
Anion binding	3.78911E-40	1214	10185	0.12	5.49084E-39	1460	10194	0.14
Nucleoside phosphate binding	1.54706E-29	1146	10050	0.11	9.10189E-29	1386	10063	0.14
Nucleotide binding	1.54706E-29	1146	10050	0.11	9.10189E-29	1386	10063	0.14
Carbohydrate derivative binding	5.78248E-33	1085	9222	0.12	1.77029E-32	1309	9227	0.14
Ribonucleotide binding	4.20887E-29	1057	9142	0.12	3.54818E-29	1281	9150	0.14
Purine nucleotide binding	6.41862E-25	1026	9059	0.11	2.06094E-24	1241	9057	0.14
Purine ribonucleotide binding	1.32576E-25	1025	9007	0.11	1.36574E-24	1236	9004	0.14
Purine ribonucleoside triphosphate binding	2.99324E-26	1008	8791	0.11	9.15765E-26	1218	8789	0.14
Adenyl ribonucleotide binding	2.8634E-36	1005	8233	0.12	2.24937E-35	1205	8230	0.15
Adenyl nucleotide binding	2.09804E-35	1005	8275	0.12	3.8074E-35	1209	8273	0.15
ATP binding	3.43182E-37	988	8017	0.12	6.44014E-37	1187	8015	0.15

Table S8 continued…

Molecular function[2]	WT[1] FDR[3]	Number of F. graminearum-regulated transcripts	Number of transcripts in the reference[4]	Transcript ratio[5]	OE[1] FDR[3]	Number of F. graminearum-regulated transcripts	Number of transcripts in the reference[4]	Transcript ratio[5]
Catalytic activity. acting on a protein	5.15726E-13	905	8608	0.11	5.94669E-17	1137	8607	0.13
Cation binding	1.93858E-13	900	8518	0.11	9.16643E-10	1067	8527	0.13
Metal ion binding	3.65553E-13	891	8443	0.11	2.5835E-09	1054	8451	0.12
Transferase activity. transferring phosphorus-containing groups	2.24833E-45	764	5497	0.14	1.63909E-49	920	5459	0.17
Kinase activity	2.5501E-61	749	4870	0.15	1.71939E-68	902	4836	0.19
Oxidoreductase activity	1.17401E-63	730	4637	0.16	2.06186E-61	854	4652	0.18
Phosphotransferase activity. alcohol group as acceptor	6.7187E-67	693	4234	0.16	7.81509E-76	837	4216	0.20
Protein kinase activity	2.70315E-81	666	3692	0.18	1.44822E-94	809	3685	0.22
Protein serine/threonine kinase activity	2.11677E-82	515	2489	0.21	8.02568E-92	613	2483	0.25
Transition metal ion binding	2.51674E-09	358	3082	0.12	3.83715E-08	427	3099	0.14
Transporter activity	8.94135E-07	358	3254	0.11	0.000155799	416	3252	0.13
Transmembrane transporter activity	3.24984E-06	341	3117	0.11	5.57135E-05	404	3113	0.13
Heme binding	7.37426E-72	281	1013	0.28	9.82412E-72	318	1019	0.31
Tetrapyrrole binding	8.11297E-62	281	1121	0.25	1.03041E-60	318	1125	0.28
Transcription regulator activity	0.021726873	250	2497	0.10	8.47978E-08	355	2519	0.14
DNA-binding transcription factor activity	1.1892E-05	240	2105	0.11	1.6038E-13	337	2125	0.16
Glycosyltransferase activity	1.43463E-10	237	1817	0.13	1.59886E-14	302	1822	0.17
Carbohydrate binding	6.80016E-58	227	821	0.28	2.34363E-60	262	828	0.32
Monooxygenase activity	3.39211E-47	217	870	0.25	1.34779E-43	241	877	0.27

Table S8 continued…

Molecular function[2]	WT[1] FDR[3]	Number of *F. graminearum*-regulated transcripts	Number of transcripts in the reference[4]	Transcript ratio[5]	OE[1] FDR[3]	Number of *F. graminearum*-regulated transcripts	Number of transcripts in the reference[4]	Transcript ratio[5]
Oxidoreductase activity. acting on paired donors. with incorporation or reduction of molecular oxygen	8.49149E-39	209	922	0.23	1.15248E-35	234	931	0.25
Lyase activity	2.93857E-21	209	1231	0.17	1.0577E-17	232	1223	0.19
Active transmembrane transporter activity	8.64757E-16	207	1357	0.15	4.56094E-14	239	1365	0.18
Iron ion binding	2.04436E-36	204	920	0.22	1.6274E-35	234	933	0.25
UDP-glycosyltransferase activity	1.88562E-23	171	883	0.19	1.3863E-26	204	873	0.23
Acyltransferase activity	2.24592E-08	168	1252	0.13	5.94282E-08	198	1249	0.16
Acyltransferase activity. transferring groups other than amino-acyl groups	1.47284E-10	165	1145	0.14	7.07789E-11	196	1139	0.17
Hydrolase activity. acting on glycosyl bonds	0.00255705	150	1338	0.11	0.012515273	177	1344	0.13
Calcium ion binding	1.63308E-14	145	860	0.17	1.02107E-13	167	859	0.19
Transferase activity. transferring alkyl or aryl (other than methyl) groups	1.37113E-36	140	496	0.28	4.0074E-39	164	506	0.32
Hydrolase activity. Hydrolyzing O-glycosyl compounds	0.005549895	131	1165	0.11	0.018769882	155	1171	0.13
Protein threonine kinase activity	8.86198E-14	125	713	0.18	2.54676E-13	145	717	0.20
Protein serine kinase activity	8.86198E-14	125	713	0.18	2.54676E-13	145	717	0.20
Glutathione transferase activity	7.50998E-62	121	242	0.50	9.12866E-68	140	250	0.56

[1] OE = TaNACL-D1-overexpressing cv. Fielder; WT = wild type.

[2] Due to many upregulated molecular processes (>100 in both genotypes), only the top 50 of those that were the most enriched are listed in the table.

[3] FDR = False discovery rate (Fischer’s exact test, FDR <0.05).

[4] Reference = The number of all expressed transcripts associated with the molecular function in the corresponding genotype.
[5] Transcript ratio = The number of *F. graminearum*-regulated transcripts associated with the molecular function relative to the total number of expressed transcripts associated with the same molecular function in the corresponding genotype.

Table S2 The transcripts that were significantly *Fusarium*-regulated in the TaNACL-D1-overeexpressing cv. Fielder (OE) but not in the wild type (WT), and that were present at significantly higher or lower levels in the pathogen-treated OE versus WT, and that had functionally characterised *Arabidopsis thaliana* top BLAST hits.

Transcript ID	Arabidopsis top BLAST hit	E-value	% Identity	Proven function of the Arabidopsis homolog[a]	Reference
TraesCS3D02G340500.1<br>TraesCS3B02G378600.1<br>TraesCS3D02G342100.1<br>TraesCS3B02G380400.1	AT5G50260.1	3.0339E-170	74	Tapetal programmed cell death, pollen development, secondary cell wall thickening during xylem development, resistance against biotrophic pathogen	[69–71]
TraesCS3D02G411200.5	AT3G43920.2	0	59	Positively regulates flowering time; Inactivated by the viral silencing suppressor protein 2b of the cucumber mosaic virus	[100, 101]
TraesCS6B02G176700.1	AT5G25060.1	0	77	phytochrome B signalling	[102]
TraesCS4B02G131700.2	AT5G13750.1	8.1704E-163	65	Regulates stomatal movements and polar auxin transport by modulating potassium and proton fluxes	[103]
TraesCS2D02G294600.1	AT5G04820.1	1.00294E-29	51	A negative regulator of floral initiation	[104]
TraesCS6A02G109100.2	AT4G31120.1	0	82	A positive regulator of floral initiation	[105]
TraesCS5D02G383900.3	AT4G21710.1	0	96	Development of embryo	[106]
TraesCS1B02G431800.5	AT5G04430.1	1.04799E-65	61	Negative regulator of tomato mosaic virus multiplication	[34]
TraesCS2B02G556100.3	AT1G50840.1	0	79	DNA replication	[107]
TraesCS2D02G396500.2	AT1G12520.1	1.9134E-119	80	Negative regulator of heat stress-responsive genes and thermotolerance	[108]
TraesCS7B02G322500.2	AT2G28880.1	0	72	Biosynthesis of PABA	[109]
TraesCS2B02G603800.1	AT5G04810.1	0	81	Embryo development; acts in the plastid nucleoids as the factor responsible of *rps12* intron 1 trans-splicing and, indirectly, in the assembly of 70S ribosomes and plastid translation.	[110, 111]
TraesCS3B02G367800.1	AT3G45100.3	8.291E-158	89	Involved in the biosynthesis of GPI; *loss of function* reduced pollen germination and tube growth, which are associated with abnormal callose deposition.	[112]
TraesCS5D02G112200.1	AT1G80570.3	7.31835E-64	53	Regulates the proteolysis of cell cycle transcription factors and connects auxin signalling with cell division; bound and activated by auxin.	[113]

Table S9 continued…

Transcript ID	Arabidopsis top BLAST hit	E-value	% Identity	Proven function of the Arabidopsis homolog[a]	Reference
TraesCS4A02G151000.1	AT1G78300.1	1.72344E-95	70	Interacts with THRUMIN1 in light-dependent chloroplast movements; Interacts with ABF3 in the nucleus; negative regulator of EOL2 (ethylene biosynthesis) protein stability; positively regulates ACS (ethylene biosynthesis) protein stability via the 26S proteasome–dependent pathway	[114–116]
TraesCS1B02G288600.2	AT1G32540.1	9.60995E-49	90	Positive regulator of cell death	[73]
TraesCS7D02G103000.1	AT1G67730.1	2.00125E-59	65	Required for the elongation of fatty acids precursors of sphingolipids, triacylglycerols, cuticular waxes and suberin; Embryo development; Interacts with a cell death suppressor and positive regulator of stress resistance BI-1.	[117, 118]
TraesCS2D02G043300.1	AT5G54650.2	1.3648E-163	69	Promotes actin polymerization; pollen germination	[119, 120]

[a] PABA = para-aminobenzoic acid; RSP12 = ribosomal protein S 12; GPI = glycosylphosphatidylinositol; ABF3 = abscisic acid-insensitive 5-like protein 6 ; EOL2 = ETO-1 like 2; ACS = acetyl-coA synthetase; BI-1 = Bax inhibitor 1.

Table S10 The transcripts that were significantly *Fusarium*-regulated in the TaNACL-D1-overeexpressing cv. Fielder (OE) but not in the wild type (WT), and that were present at significantly higher or lower levels in the pathogen-treated OE versus WT, and that had functionally characterised rice *(Oryza sativa)* top BLAST hits.

Transcript ID	Rice top BLAST hit	E-value	% Identity	Proven function of rice homolog[a]	Reference
TraesCS3D02G340500.1; TraesCS3B02G378600.1	Os08t0556900-01	0	89	Negatively regulates stress-induced cell death	[72]
TraesCS3D02G342100.1; TraesCS3B02G380400.1	Os08t0556900-02	0	89	Negatively regulates stress-induced cell death	[72]
TraesCS3D02G411200.5	Os01t0909200-01	0	79	Directly targets genes involved in gibberellin and brassinosteroid homeostasis; Silencing caused dwarfism, larger flag leaf angle, and fewer secondary branches; phased small RNA biogenesis	[121, 122]
TraesCS4B02G131700.2	Os12t0133100-02	0	72	Involved in sequestering a phytosiderophore DMA into the vacuoles. Knockout enhanced accumulation of Fe and Zn in polished rice grains through DMA-increased solubilization of Fe and Zn deposited in the node	[123]
TraesCS2B02G556100.3	Os08t0175300-01	0	75	Plastidial DNA replication and repair	[124]
TraesCS2B02G603800.1	Os04t0684500-01	0	95	Chloroplast biogenesis in rice under cold stress; intron splicing of rpl2 and rps12 in chloroplasts	[125]
TraesCS4A02G151000.1	Os11t0546900-01	2.1E-113	78	Interacts with BZR1 (brassinosteroid signal transduction in rice); Interacts with ABI5 (involved in ABA signalling, growth, and abiotic stress responses)	[126, 127]
TraesCS1B02G288600.2	Os08t0159500-01	1E-54	96	Negative regulator of PCD and defence against virulent blast fungus; and positive regulator of callus differentiation and tolerance to fumonisin 1	[74]
TraesCS2D02G176200.3	Os07t0616000-01	0	78	Silencing negatively affected grain size due to reduction of cell number and cell size, and increased stem thickness and number of vascular bundles	[128, 129]
TraesCS7D02G001300.1	Os12t0247700-01	5.08E-70	57	Quantitative broad-spectrum resistance against different pathogens including bacteria, oomycetes, and fungi	[130]

[a] DMA = 29-deoxymugineic acid; BZR1 = brassinazole-resistant 1; ABI5 = ABA insensitive 5; ABA = abscisic acid; PCD = programmed cell death; Fe = iron; Zn = zinc.

Table S11 The transcripts that were present at significantly higher or lower levels in the non-treated (day zero) *TaNACL-D1*-overexpressing cv. Fielder as compared to the wild type, and that were associated with development-related biological processes.

Transcript ID	Fold change	Description	Biological processes
TraesCS2D02G387600.1	0.01	Probable metal-nicotianamine transporter YSL9	Response to iron ion; siderophore-dependent iron import into cell; seed development; transmembrane transport
TraesCS3B02G054400.1	0.13	E3 ubiquitin-protein ligase SINA-like 10	Ubiquitin-dependent protein catabolic process; multicellular organism development; protein ubiquitination
TraesCS6A02G312500.2	0.01	Probable histidine kinase 6	Phosphorylated signal transduction system; protein dephosphorylation; osmosensory signalling pathway; sulfate transport; nucleoside metabolic process; response to water deprivation; cytokinin-activated signalling pathway; regulation of seed germination; embryonic root morphogenesis; cellular response to phosphate starvation; peptidyl-histidine phosphorylation; carbohydrate homeostasis; defence response to bacterium; regulation of meristem development; regulation of shoot system development; cellular response to sucrose stimulus
TraesCS6B02G183600.1	0.02	Succinate-semialdehyde dehydrogenase, mitochondrial	Cellular aldehyde metabolic process; glutamate decarboxylation to succinate; response to heat; response to cold; response to light stimulus; gamma-aminobutyric acid catabolic process; adaxial/abaxial axis specification; maintenance of shoot apical meristem identity; cotyledon development; reactive oxygen species metabolic process; response to salt
TraesCS7A02G053900.1	0.50	Predicted protein	Embryo development ending in seed dormancy
TraesCS7A02G371200.3	0.02	Protein argonaute PNH1	Stem cell population maintenance; gene silencing by RNA; leaf development
TraesCS7B02G021100.1	<0.01	MADS-box transcription factor 5	Flower development; cell differentiation; positive regulation of transcription by RNA polymerase II
TraesCS1D02G162600.2	128	Protein YABBY 3	Polarity specification of adaxial/abaxial axis; fruit development; abaxial cell fate specification; regulation of shoot apical meristem development; regulation of leaf development
TraesCS3B02G224600.3	128	DNA replication licensing factor MCM4	Double-strand break repair via break-induced replication; pre-replicative complex assembly involved in nuclear cell cycle DNA replication; DNA unwinding involved in DNA replication; DNA strand elongation involved in DNA replication; pollen development; mitotic DNA replication initiation
TraesCS5D02G066200.1	2	Probable DNA helicase MCM8	Double-strand break repair via homologous recombination; male meiotic nuclear division; female meiotic nuclear division; pollen development; DNA duplex unwinding

Table S12 The transcripts that were present at significantly higher or lower levels in the TaNACL-D1-overexpressing cv. Fielder as compared to the wild type irrespective of the treatment and that had functionally characterised *Arabidopsis* top BLAST hits.

Transcript ID	Arabidopsis top BLAST hit	E-value	% Identity	Proven function of Arabidopsis homolog[1]	Reference
TraesCS5D02G111300.1	AT1G02250.1	2.39712E-7	42	Xylem development	[36]
TraesCS5A02G259000.1	AT1G06390.2	8.71406E-25	91	Brassinosteroid signalling and salt tolerance	[41, 131]
TraesCS1B02G005000.1	AT1G09730.2	7.83879E-4	50	Floral initiation, flower development, embryo development and gametogenesis, ABA signalling	[37, 38, 132]
TraesCS5B02G104200.1	AT1G02250.1	4.1898E-9	39	Xylem development	[36]
TraesCS4A02G469500.1	AT2G03200.1	3.82748E-75	51	Primary and lateral root development	[39]
TraesCS2B02G042700.2	AT3G14460.1	9.60435E-77	56	Defence against biotrophic and hemibiotrophic plant pathogens	[44]
TraesCS3D02G349200.1	AT3G53520.1	0.0	80	Biosynthesis of UDP-xylose	[42]
TraesCS6A02G330100.1	AT1G44800.1	3.85612E-125	70	Amino acid homeostasis in siliques	[45]
TraesCS1B02G361800.1	AT1G06390.2	8.71406E-25	91	Brassinosteroid signalling and salt tolerance	[41, 131]
TraesCS7B02G375300.1	AT1G79550.2	0.0	91	Glycolysis	[43]

[1] ABA = abscisic acid; UDP = uridine diphosphate.

Table S13 The transcripts that were present at significantly higher or lower levels in the TaNACL-D1-overexpressing cv. Fielder as compared to the wild type irrespective of the treatment and that had functionally characterised rice (*Oryza sativa*) top BLAST hits.

Transcript ID	Rice top BLAST hit	E-value	% Identity	Proven function of rice homolog	Reference
TraesCS5A02G259000.1	Os05t0207500-01	2.45862E-26	89	Negative regulator of brassinosteroid (BR) signalling	[40]
TraesCS4A02G156500.1	Os03t0397400-01	0.0	95	Ca^{2+} transport	[46]
TraesCS1B02G361800.1	Os05t0207500-01	2.13037E-26	89	Negative regulator of brassinosteroid signalling	[40]

SUPPLEMENTAL FIGURES

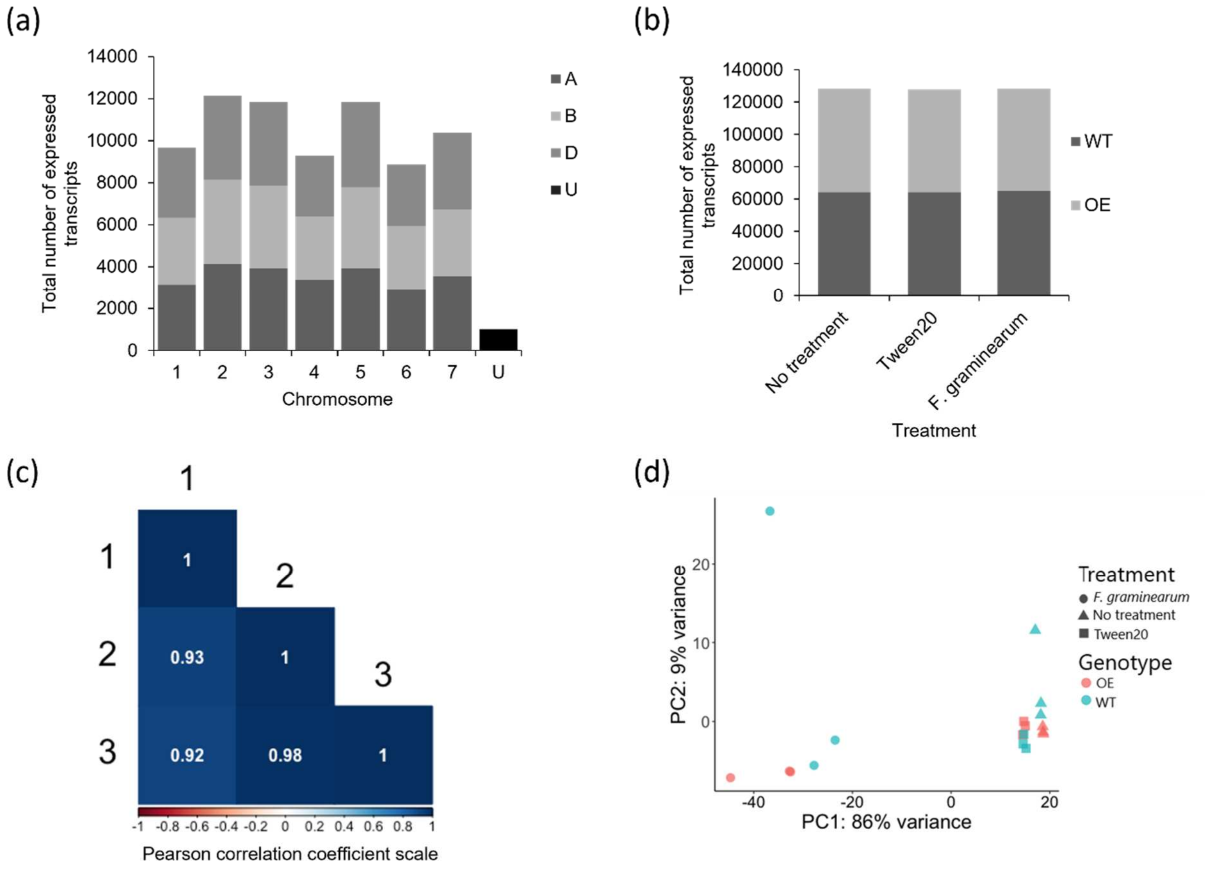

Figure S1 Quality assessment of RNA transcriptomes from 18 sequenced samples: **(a)** distribution of expressed transcripts across wheat subgenomes (A, B, D) and chromosomes (1-7), including an unknown subgenome/chromosome U; **(b)** number of expressed transcripts within each treatment x genotype combination; **(c)** the Pearson's correlation of expressed transcripts between three trials. The correlation coefficient within each box represents each pairwise correlation; and **(d)** principal component (PC) plot of variance-stabilised-transformed read counts across 18 samples. Circles, triangles, and squares indicate *Fusarium graminearum* treatment, no-treatment control (day zero) and Tween 20 treatment (mock), respectively. Colours refer to the genotypes: OE (overexpressing line) and WT (wild type).

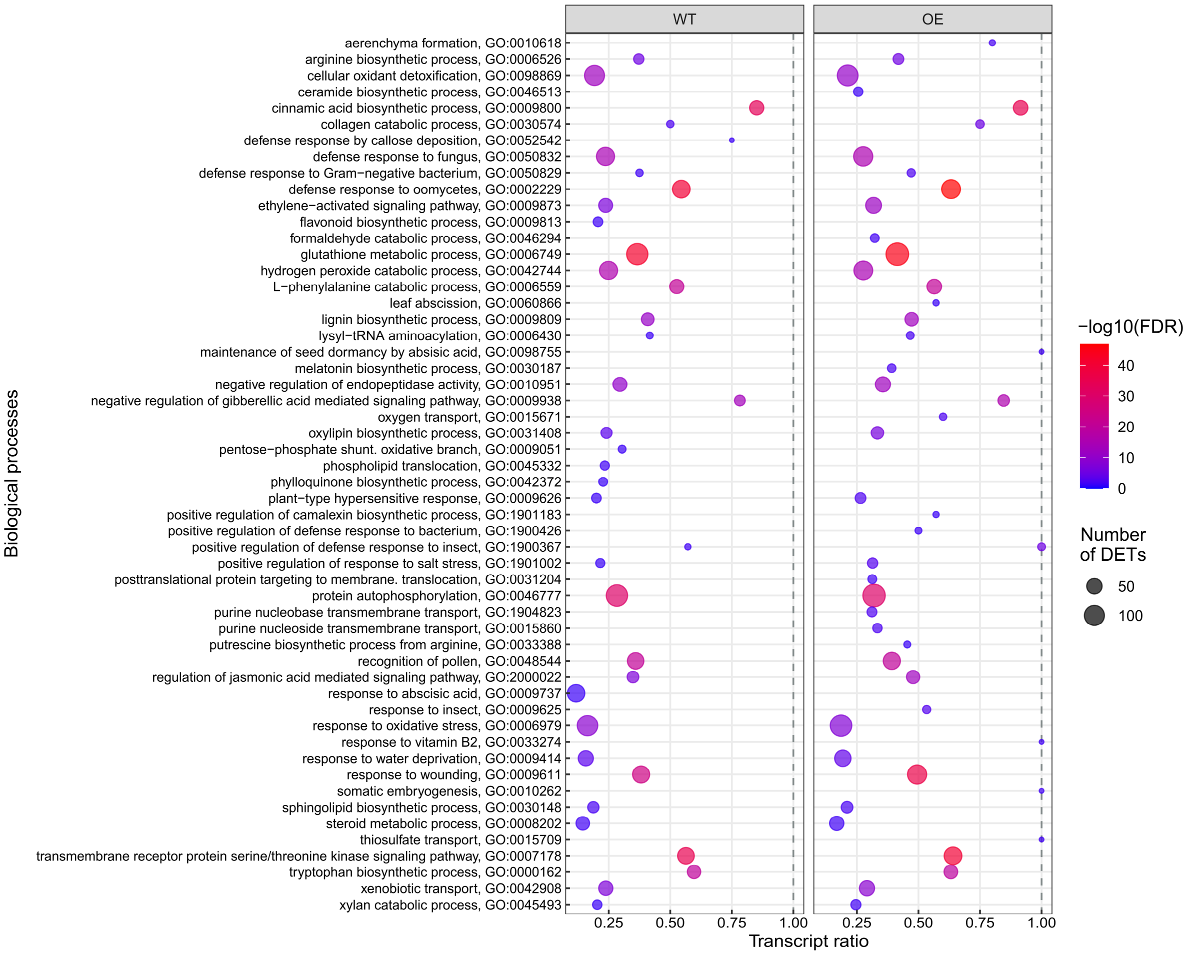

Figure S2 Descent biological processes associated with *Fusarium graminearum*-upregulated transcripts in the TaNACL-D1-overexpressing (OE) wheat cv. Fielder and the WT at one day post-inoculation. The coloured scale on the right represents negative logarithmic scale with a base 10 of the false discovery rate (FDR) values for each of these processes. Abbreviation: DETs, differentially expressed transcripts.

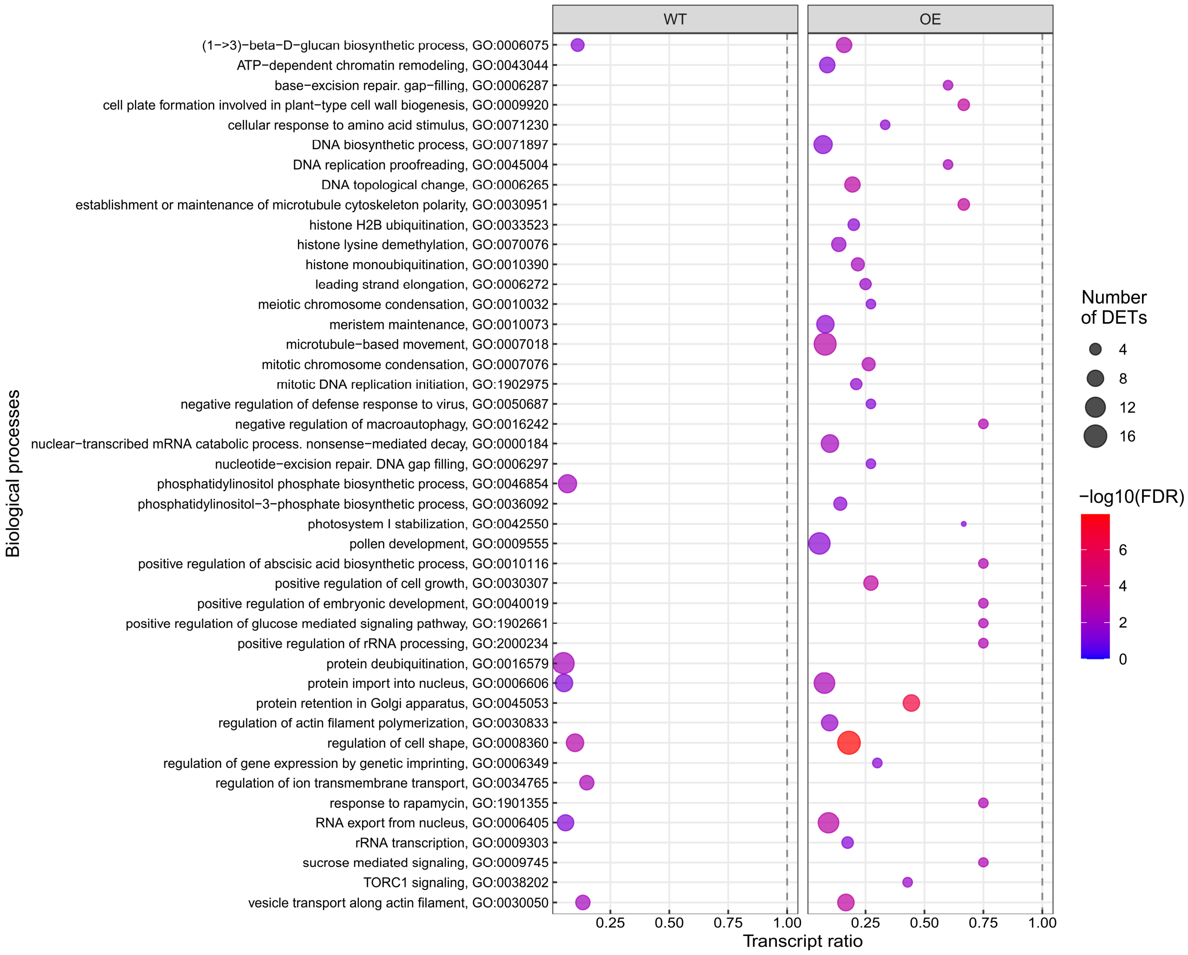

Figure S3 Descent biological processes associated with *Fusarium graminearum*-downregulated transcripts in the TaNACL-D1-overexpressing (OE) wheat cv. Fielder and the wild type (WT) at one day post-inoculation. The coloured scale on the right represents negative logarithmic scale with a base 10 of the false discovery rate (FDR) values for each of these processes. Abbreviation explanation: DETs = differentially expressed transcripts.

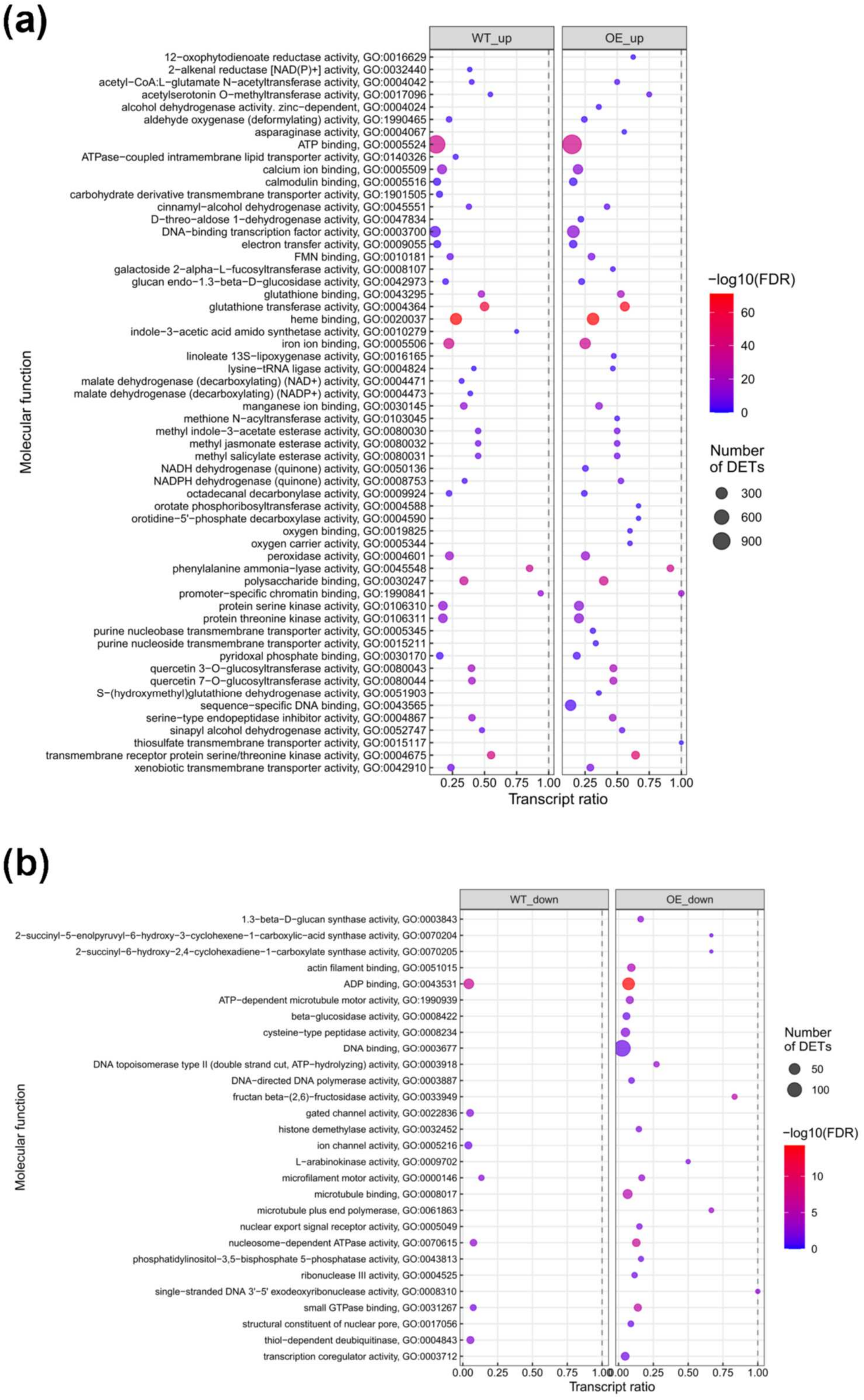

Figure S4 Descent molecular functions significantly enriched with associated: **(a)** *Fusarium graminearum*-upregulated; and **(b)** *F. graminearum*-downregulated transcripts in the TaNACL-D1-overexpressing cv. Fielder (OE) and the wild type (WT). The coloured scale on the right represents negative logarithmic scale with a base 10 of the false discovery rate (FDR) values for each of these molecular functions. Abbreviations explanation: up = upregulated; down = downregulated; DETs = differentially expressed transcripts.

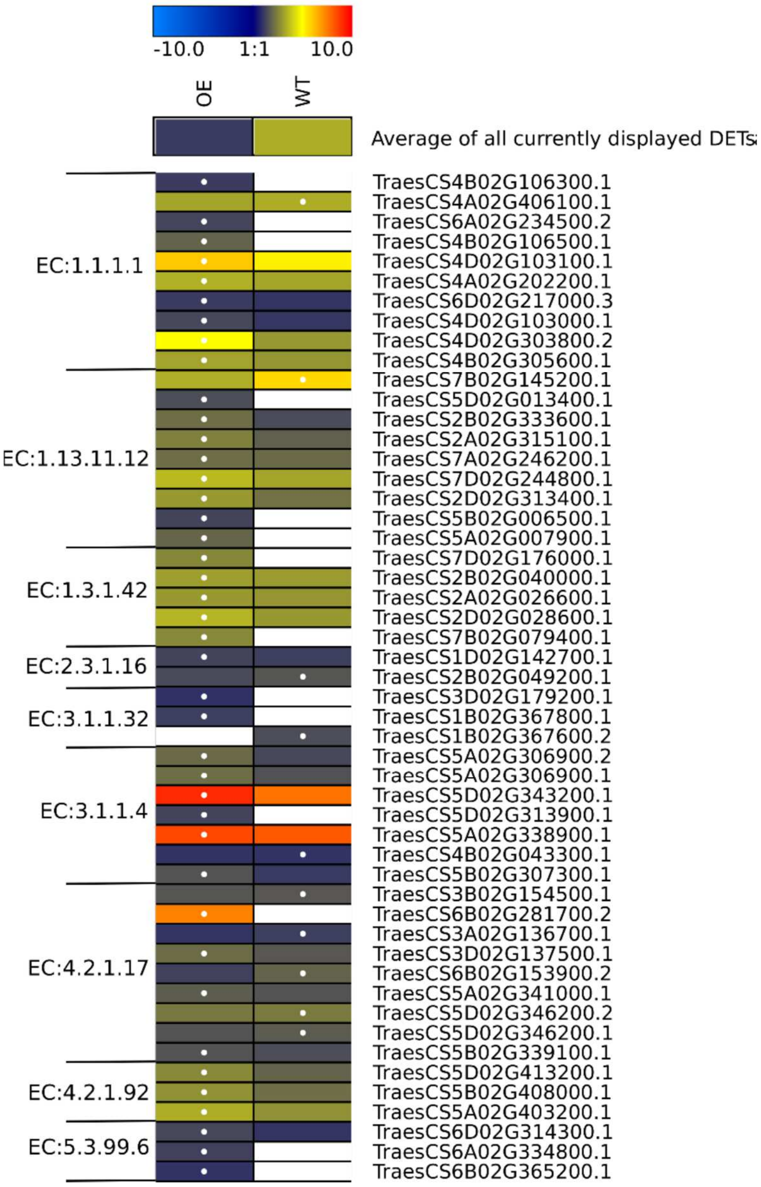

Figure S5 The heatmap of the *F. graminearum*-upregulated transcripts associated with alpha-linolenic acid metabolism at one day post-inoculation in the TaNACL-D1-overexpressing cv. Fielder (OE) and the wild type (WT). Enzyme codes: 1.1.1.1 = Alcohol dehydrogenase; 1.3.11.12 = linoleate 13S-lipoxygenase; 1.3.1.42 = 12-oxophytodienoate reductase; 2.3.1.16 = acetyl-CoA C-acyltransferase; 3.1.1.32 = phospholipase A1; 3.1.1.4 = phospholipase A2; 4.2.1.17 = enoyl-CoA hydratase; 4.2.1.92 = hydroperoxide dehydratase; 5.3.99.6 = allene-oxide cyclase. The coloured scale on the top represents log2 fold change. The white fields represent transcripts that are not regulated by the fungus. A dot within a coloured field indicates that the expression of the transcript in that genotype differed as compared to the other genotype. Abbreviation: DETs, differentially expressed transcripts.

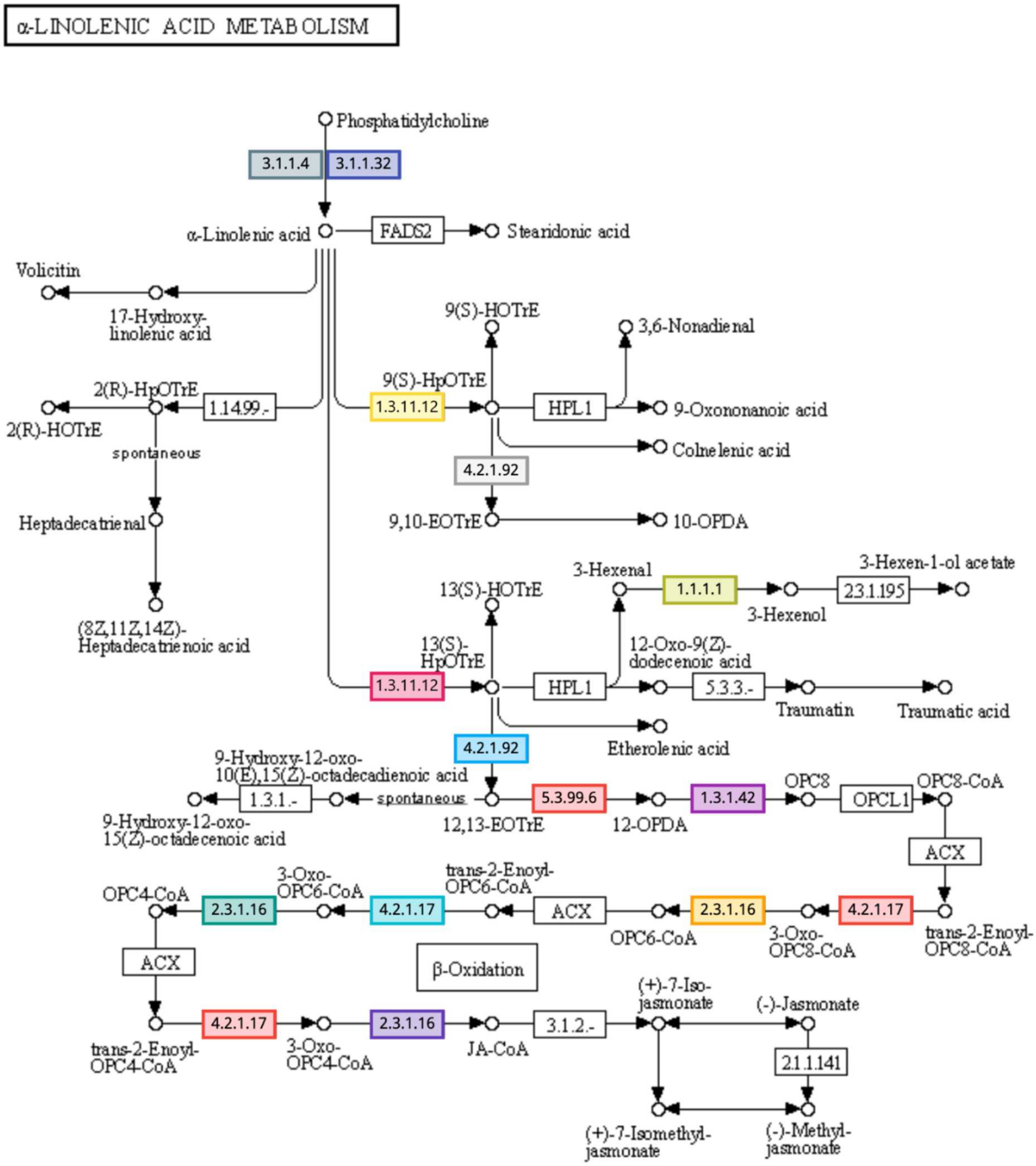

Figure S6 The map of the alpha-Linolenic acid metabolism KEGG pathway (map00592) [133]. The coloured enzyme codes are associated with the *F. graminearum*-upregulated transcripts at one day post-inoculation in both the TaNACL-D1-overexpressing cv. Fielder and the wild type. Enzyme codes: 1.1.1.1 = alcohol dehydrogenase1.3.11.12 = linoleate 13S-lipoxygenase;.1.3.1.42 = 12-oxophytodienoate reductase; 2.3.1.16 = acetyl-CoA C-acyltransferase; 3.1.1.32 = phospholipase A1; 3.1.1.4 = phospholipase A2; 4.2.1.17 = enoyl-CoA hydratase; 4.2.1.92 = hydroperoxide dehydratase; 5.3.99.6 = allene-oxide cyclase.

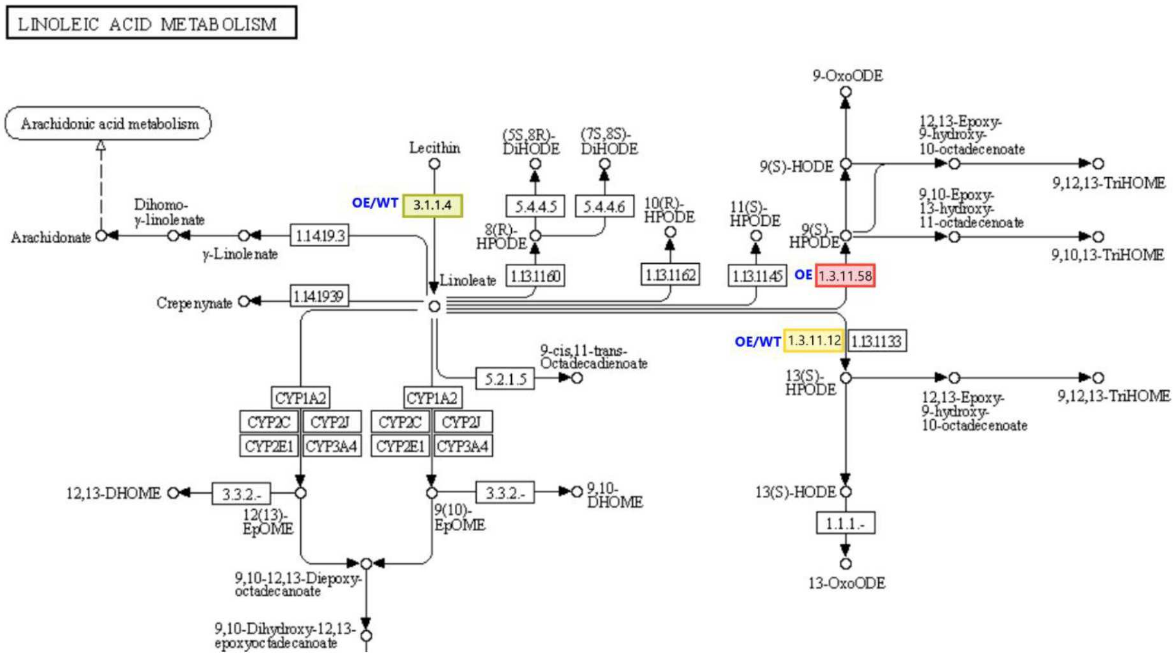

Figure S7 The map of the linoleic acid metabolism KEGG pathway (map00591) [133]. The coloured enzyme codes are associated with the *F. graminearum*-upregulated transcripts at one day post inoculation in only the TaNACL-D1-overexpressing cv. Fielder (OE) or in both the OE and the wild type (OE/WT) (highlighted with blue font). Enzyme codes: 1.3.11.12 = linoleate 13S-lipoxygenase; 1.3.11.58 = linoleate 9S-lipoxygenase; 3.1.14 =phospholipase A(2).

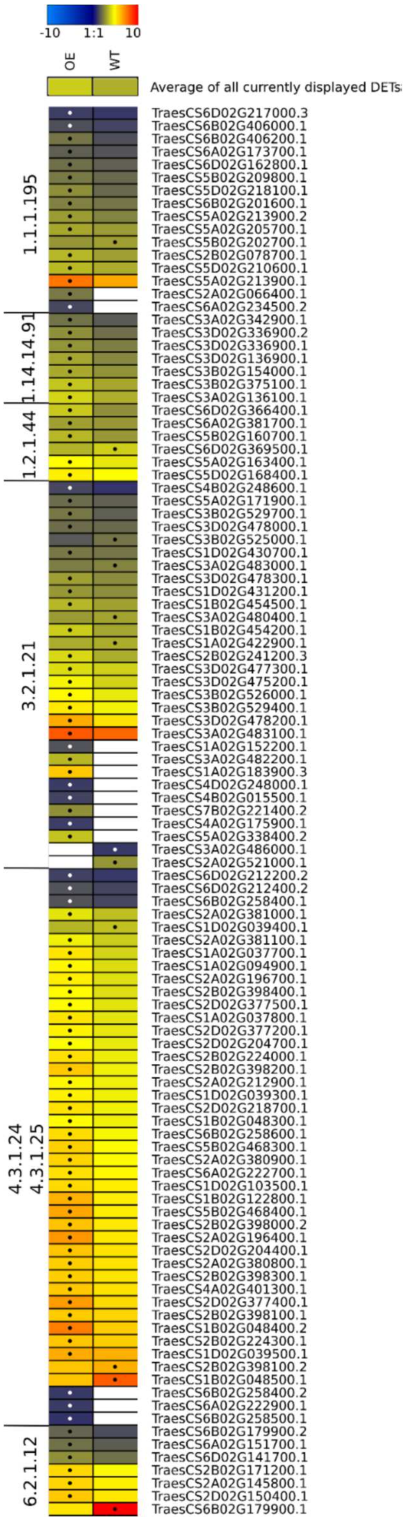

Figure S8 The heatmap of the *F. graminearum*-upregulated transcripts associated with the phenylpropanoid biosynthesis at one day post-inoculation in the *TaNACL-D1*-overexpressing cv. Fielder (OE) and the wild type (WT). Enzyme codes: 1.1.1.195 = cinnamyl alcohol dehydrogenase; 1.4.14.91 = Trans-cinnamate 4-monooxygenase; 1.2.1.44 = Cinnamoyl-CoA reductase; 3.2.1.21 = beta-glucosidase; 4.3.1.24/25 = phenylalanine/tyrosine ammonia-lyase ; 6.2.1.12 = 4-coumarate—CoA ligase. The coloured scale on the top represents log2 fold change. The white fields represent transcripts that are not regulated by the fungus. A dot within a coloured field indicates that the expression of the transcript in that genotype differed as compared to the other genotype. Abbreviation explanation: DETs; differentially expressed transcripts.

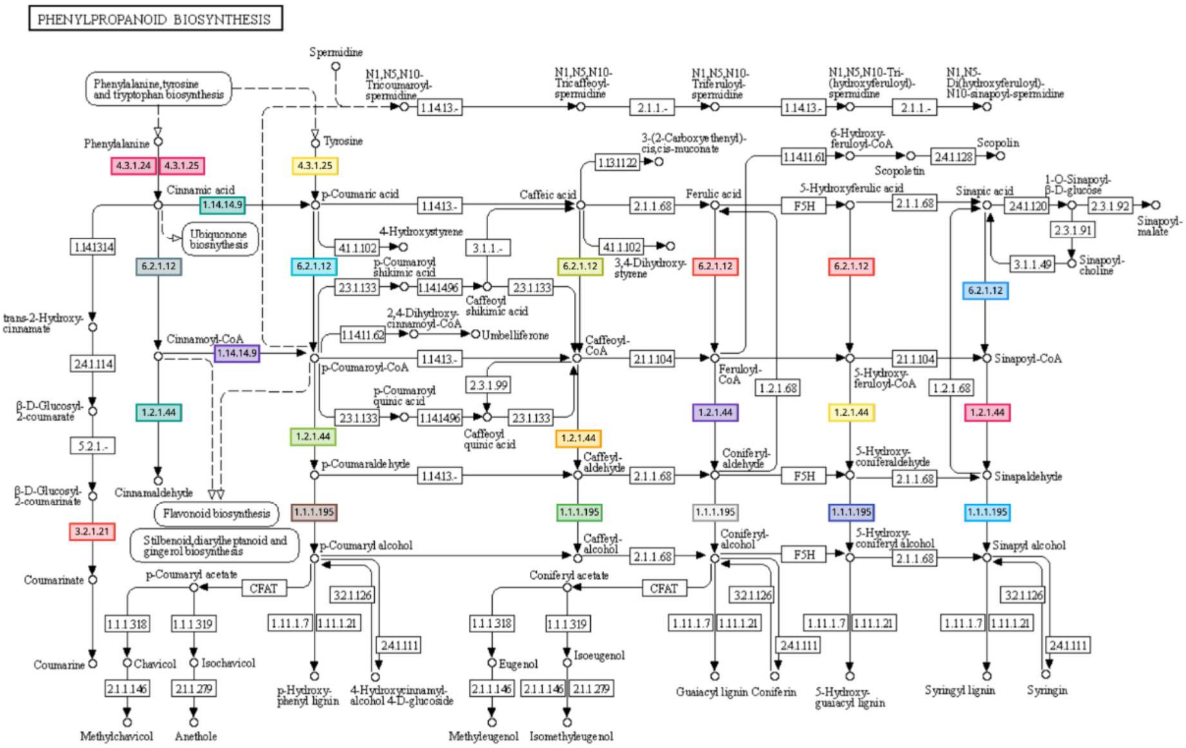

Figure S9 The map of the phenylpropanoid biosynthesis KEGG pathway (map00940) [133]. The coloured enzyme codes are associated with the *F. graminearum*-upregulated transcripts associated with phenylpropanoid biosynthesis at one day post-inoculation in the TaNACL-D1-overexpressing cv. Fielder (OE) and in the wild type (WT). Enzyme codes explanation: 1.1.1.195 = cinnamyl alcohol dehydrogenase; 1.4.14.91 = trans-cinnamate 4-monooxygenase; 1.2.1.44 = C=cinnamoyl-CoA reductase; 3.2.1.21 = beta-glucosidase; 4.3.1.24/25 = phenylalanine/tyrosine ammonia-lyase; 6.2.1.12 = 4-coumarate--CoA ligase.
